# Strongly Anisotropic Strain‐Tunability of Excitons in Exfoliated ZrSe_3_

**DOI:** 10.1002/adma.202103571

**Published:** 2021-10-15

**Authors:** Hao Li, Gabriel Sanchez‐Santolino, Sergio Puebla, Riccardo Frisenda, Abdullah M. Al‐Enizi, Ayman Nafady, Roberto D'Agosta, Andres Castellanos‐Gomez

**Affiliations:** ^1^ Materials Science Factory Instituto de Ciencia de Materiales de Madrid (ICMM‐CSIC) Madrid E‐28049 Spain; ^2^ GFMC Departamento de Física de Materiales & Instituto Pluridisciplinar Universidad Complutense de Madrid Madrid 28040 Spain; ^3^ Department of Chemistry College of Science King Saud University Riyadh 11451 Saudi Arabia; ^4^ Nano‐Bio Spectroscopy Group and European Theoretical Spectroscopy Facility (ETSF) Departamento de Polímeros y Materiales Avanzados: Física Química y Tecnología Universidad del País Vasco UPV/EHU Avenida Tolosa 72 San Sebastián E‐20018 Spain; ^5^ IKERBASQUE Basque Foundation for Science Plaza Euskadi 5 Bilbao E‐48009 Spain

**Keywords:** 2D materials, anisotropy, band‐gap engineering, strain engineering, zirconium triselenide (ZrSe _3_)

## Abstract

The effect of uniaxial strain on the band structure of ZrSe_3_, a semiconducting material with a marked in‐plane structural anisotropy, is studied. By using a modified three‐point bending test apparatus, thin ZrSe_3_ flakes are subjected to uniaxial strain along different crystalline orientations monitoring the effect of strain on their optical properties through micro‐reflectance spectroscopy. The obtained spectra show excitonic features that blueshift upon uniaxial tension. This shift is strongly dependent on the direction along which the strain is being applied. When the flakes are strained along the *b*‐axis, the exciton peak shifts at ≈60–95 meV %^−1^, while along the *a*‐axis, the shift only reaches ≈0–15 meV %^−1^. Ab initio calculations are conducted to study the influence of uniaxial strain, applied along different crystal directions, on the band structure and reflectance spectra of ZrSe_3_, exhibiting a remarkable agreement with the experimental results.

## Graphical abstract

Zirconium triselenide is a 2D semiconductor material with an interesting in‐plane anisotropic crystal structure. Here, it is demonstrated that the excitons of this material are very sensitive to uniaxial strains and, interestingly, their tunability strongly depends on the direction along which the uniaxial strain is applied.

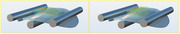

## 1 Introduction

Applying mechanical deformations has become a powerful approach to modify the vibrational, optical, and electronic properties of 2D materials.^[^ ^1^, ^2^, ^3^, ^4^, ^5^, ^6^, ^7^ ^]^ In principle, 2D materials can sustain unprecedented strains without breaking.^[^ ^8^, ^9^, ^10^ ^]^ Moreover, their band structures are rather strain‐sensitive,^[^ ^11^, ^12^, ^13^, ^14^ ^]^ making them very suitable for strain engineering applications. In addition, 2D materials offer the possibility to apply strain in various ways (e.g., uniaxial/biaxial, homogenous/inhomogenous), which can be adjusted by the end‐user at will. Strain engineering in conventional 3D materials, in contrast, typically relies on forcing the epitaxial growth of a material onto a substrate with a given lattice parameter mismatch, providing a fixed strain that cannot be adjusted after growth. Among the different strategies to strain engineer 2D materials, the application of uniaxial strain through bending flexible substrates with a bending jig apparatus is one of the most popular approaches.^[^ ^1^, ^11^, ^12^, ^15^, ^16^, ^17^, ^18^ ^]^ This method presents some issues when applied to 2D materials with in‐plane anisotropic properties. Indeed, the effect of uniaxial strain along different crystal orientations is expected to modify the properties of these anisotropic 2Ds differently. Despite the recent interest on these families of anisotropic 2D materials,^[^ ^19^, ^20^, ^21^, ^22^, ^23^, ^24^, ^25^, ^26^ ^]^ the number of reported research works focused on studying the effect of strain along different crystal directions is still very scarce and primarily focused on the investigation of strain tunable Raman modes in black phosphorus, PdSe_2_, or tellurium.^[^ ^27^, ^28^, ^29^, ^30^, ^31^ ^]^ The group IV–V transition metal trichalcogenides (TMTCs) are a less‐explored family of materials with quasi‐1D electrical and optical properties stemming from a reduced in‐plane structural symmetry.^[^ ^24^, ^32^, ^33^, ^34^ ^]^ These materials have a general formula of MX_3_ being M a transition metal atom belonging to either group IVB (Ti, Zr, Hf) or group VB (Nb, Ta) and X chalcogen atoms from group VIA (S, Se, Te).

**Figure 1:**
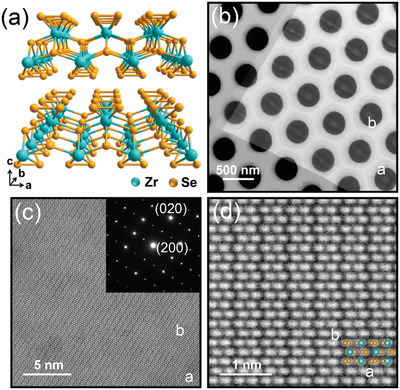
Structural characterization of ZrSe_3_. a) 3D representation of the crystal structure of ZrSe_3_ where the in‐plane anisotropy can be resolved. b,c) Low magnification and atomic resolution HAADF images of a mechanically exfoliated ZrSe_3_ flake transferred over a holey Si_3_N_4_ membrane. The inset in (c) shows an SAED pattern acquired at the same region; d) Atomic resolution HAADF image of the ZrSe_3_ flake down the (001) direction depicting the chains‐like structure along the *b*‐axis. The superimposed atomic model depicts the Zr (cyan) and Se (orange) atomic column positions.

In this work, we focused on ZrSe_3_, a semiconductor of the transition metal trichalcogenide family^[^ ^24^ ^]^ with a strong in‐plane anisotropic structure similar to that of TiS_3_,^[^ ^35^, ^36^, ^37^, ^38^ ^]^ along different crystal directions (see **Figure** **1**a). ZrSe_3_ has been scarcely studied experimentally so far. Xiong et al. presented a photodetector based on individual ZrSe_3_ nanobelts^[^ ^39^ ^]^ and Zhou et al. proposed its potential application in thermoelectrics through calculations,^[^ ^40^ ^]^ but little is still known about the fundamental electronic and optical properties of this semiconducting material. We explored how applying strain along different crystal orientations modifies the excitonic features in the optical spectra. Our straining technique relies on a three‐point bending test apparatus, and it can be easily adapted by other groups that are already working on strain engineering of 2D materials. Using this straining approach, we demonstrate how the direction along which we apply the uniaxial strain has a strong effect on the strain tunability of the excitonic features on this anisotropic 2D material. Evidently, a giant anisotropy in the strain‐induced exciton shift was found. The strain gauge factor varies from ≈60 to 95 meV %^−1^ for uniaxial strain applied along the *b*‐axis to ≈0–15 meV %^−1^ when the uniaxial strain is applied along the *a*‐axis. Additionally, ab initio calculations were performed, supporting the finding of the strongly anisotropic strain‐tunable direct band‐gap transitions observed in the experiments. Therefore, being able to accurately align the direction of the uniaxial strain axis with specific crystalline orientations is strongly appealing to further employ this family of 2D materials in strain engineering applications.

## 2 Sample Fabrication and Straining Setup

ZrSe_3_ flakes are obtained by mechanical exfoliation of bulk crystals and transferred through an all‐dry deterministic placement method^[^ ^41^, ^42^, ^43^ ^]^ (see the Experimental Section). Figure 1b shows a high‐angle annular dark‐field scanning transmission electron microscopy (HAADF‐STEM) image of a thin ZrSe_3_ flake transferred onto a holey Si_3_N_4_ TEM grid to characterize its crystal structure. Figure 1c shows an atomic resolution HAADF image of the same flake along the (001) direction, in which the anisotropic in‐plane structure of ZrSe_3_ is visible. The inset shows a selected area electron diffraction (SAED) pattern acquired at the same region corresponding to a monoclinic (*P2_1_/m*) crystal structure. A high magnification atomic resolution HAADF image shown in Figure 1d clearly illustrates the chain‐like structure of ZrSe_3_ within the *a*–*b* plane, similar to that of TiS_3_.^[^ ^24^ ^]^

**Figure 2:**
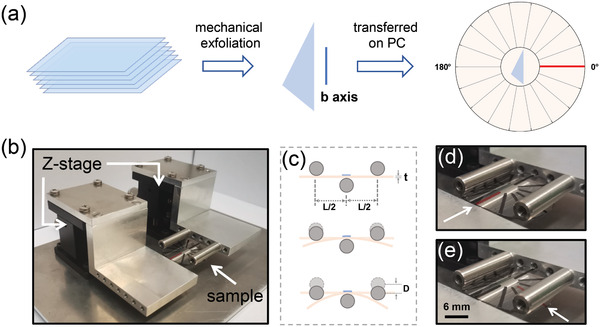
Setup for angle‐resolved uniaxial straining measurements. a) Cartoon of the fabrication process of the test sample. A disk‐shaped polycarbonate substrate is marked with a permanent marker every 20° angle from 0°–360°, highlighting the 0° with a red line. An exfoliated ZrSe_3_ flake is then transferred onto the center of the disk by an all‐dry deterministic placement method. b) Picture of the three‐points bending setup used for the strain engineering experiments with a disk‐shaped flexible substrate loaded between the pivotal points. c) Schematic representation of the three‐point bending process. d,e) Pictures of the sample upon 0.65% of uniaxial strain applied along two orthogonal directions (notice the position of the red marker line).

The angle‐dependent uniaxial strain measurements are based on the integration of disk‐shaped flexible substrates, instead of the commonly used rectangular beam, in a bending‐test apparatus. **Figure** **2**a is a schematic representation of the fabrication process. The samples are fabricated out of a disk‐shaped polycarbonate substrate (thickness = 250 µm). Permanent marker lines are drawn on the surface to guide the angle adjustment. The ZrSe_3_ flakes are then transferred onto the geometrical center of the disk surface by means of an all‐dry deterministic placement method with an accuracy of ≈10 µm.^[^ ^41^, ^42^, ^43^ ^]^

The disk‐shaped substrate with the ZrSe_3_ is then loaded in a homebuilt three‐point bending jig apparatus to apply uniaxial strain through bending of the substrate (Figure 2b,c).^[^ ^15^ ^]^ After a strain load/unload cycle, the disk‐shaped sample can be rotated to apply uniaxial strain along another direction. Figure 2d,e shows an example where uniaxial strain is applied along two orthogonal directions (notice the position of the red marker line). The amount of strain can be extracted from the geometry of the disk substrate and the bending apparatus through

(1) $$\varepsilon =\frac{6tD}{L^{2}}$$

where *t* is the thickness of the substrate, *L* is the distance between the outer pivotal points, and *D* is the deflection of the central pivotal point (see Figure 2c). Note that we have experimentally validated this formula to calculate the strain in ref. ^[^ ^15^ ^]^ by directly measuring the separation of micro‐fabricated features in the surface of the polycarbonate substrates upon controlled deflection of the substrate with the three‐point bending apparatus.

## 3 Results

**Figure 3:**
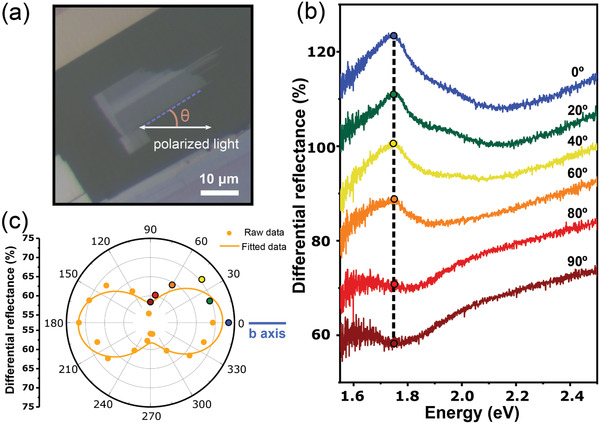
Identification of crystalline orientations of ZrSe_3_. a) Optical microscopy image of a few‐layer ZrSe_3_ flake. In the angle‐resolved measurements, θ is defined as the angle between the cleaved long straight edge of the flake (dashed blue line) and the polarization direction (parallel to the horizontal axis of the microscope, white arrow). b) Micro‐reflectance spectra of the ZrSe_3_ flake (unstrained) as a function of sample rotation angle from 0° to 90° while the incident light is linearly polarized parallel to the horizontal axis. The spectra have been vertically offset by 10% to facilitate the comparison. c) Polar plot of the differential reflectance intensity at ≈1.75 eV for different angles between the incident linearly polarized light and the cleaved long straight edge of the ZrSe_3_ flake. The color circles correspond to the spectra shown in (b).

**Figure** **3**a displays an optical microscopy image of a ≈16 nm thick ZrSe_3_ flake (see Figure S1, Supporting Information), transferred onto the center of a disk‐shaped flexible polycarbonate substrate. For the angle‐dependent uniaxial straining experiments, it is crucial to accurately determine the crystal orientation of the ZrSe_3_ flake under study. This can be done by measuring reflection or absorption spectra with linearly polarized incident light. When the incident light is linearly polarized along the *b*‐axis, the spectra show prominent excitonic features, even at room temperature.^[^ ^44^, ^45^ ^]^ When the light is polarized along the *a*‐axis, on the other hand, the intensity of these excitonic features decreases substantially.

We have used a homebuilt differential reflectance system to acquire reflectance spectra with linearly polarized incident light to determine the ZrSe_3_ crystal orientation.^[^ ^46^, ^47^ ^]^ We found that other optical spectroscopic techniques, like photoluminescence or Raman spectroscopy, could not be carried out in the ZrSe_3_ due to laser‐induced damage of the flakes even at very low laser power density values (≈40 µW µm^−2^). Figure S2 in the Supporting Information shows the damage caused by the laser when attempting to carry out Raman spectroscopy and photoluminescence measurements with low excitation power. In our differential reflectance experiments, we fixed the incident light linearly polarized parallel to the horizontal axis of the microscope while the ZrSe_3_ flake is rotated to vary the orientation between the linearly polarized light and the crystal axes. Figure 3b shows differential reflectance spectra acquired for different angles (θ) between the incident linearly polarized light and the long cleaved edge of the ZrSe_3_ flake (highlighted with a dashed blue line in Figure 3a). When the straight long edge of the flake is parallel to the linearly polarized light (labeled as 0° or 180°), the spectra show a broad peak feature at ≈1.75 eV and a shoulder at ≈1.9 eV. These peaks are attributed to the generation of excitons (called A and B in the literature, respectively) due to direct valence‐to‐conduction band transitions.^[^ ^44^, ^45^ ^]^ The intensity of these excitonic peaks decreases when the flake is rotated and it reaches its minimum intensity when the long edge of the flake is perpendicular to the incident linearly polarized light. This observation indicates that the *b*‐axis of the ZrSe_3_ flake is oriented parallel to the long edge of the flake.^[^ ^44^, ^45^ ^]^ Figure 3c represents the intensity of the A exciton peak at different angles between the incident linearly polarized light and the long straight edge of the flake. The polar plot clearly illustrates how the intensity of the exciton feature reaches its maximum value when the long edge of the flake is aligned parallel to the incident polarized light (horizontal axis).

After determining the crystal orientations of the ZrSe_3_ flake under study, we subject it to uniaxial strain cycles. Differential reflectance spectroscopy at different strain values was conducted to infer the effect of strain on the excitonic features of the optical spectra. After each uniaxial strain cycle, the disk‐shaped substrate is rotated by ≈20°, changing the angle (θ) between the crystal *b*‐axis and the uniaxial strain direction. We label as 0°/180° the situation where the uniaxial strain is parallel to the *b*‐axis and 90°/270° the one where the uniaxial strain is perpendicular to the *b*‐axis. For this experiment, the incident polarized light is kept parallel to the *b*‐axis to maximize the intensity of the exciton peaks but comparable results are obtained using unpolarized light (see Figure S4, Supporting Information).

**Figure 4:**
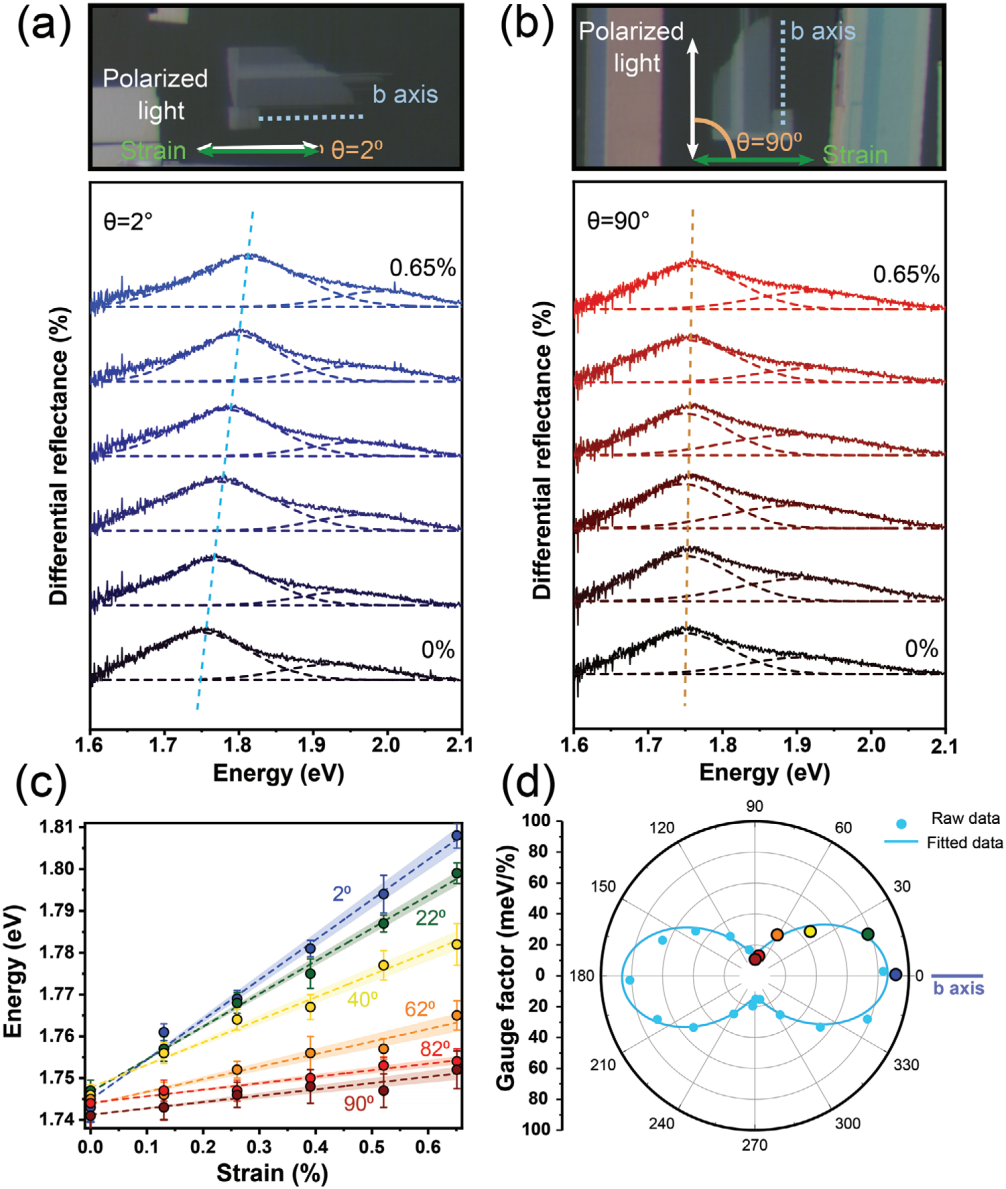
Angle‐resolved micro‐reflectance spectra of ZrSe_3_ under different uniaxial strain from 0% to 0.65%. a,b) Micro‐reflectance spectra acquired when the uniaxial strain direction is parallel to the *b*‐axis and the *a*‐axis of the ZrSe_3_ flake, respectively. c) A‐exciton peak energy as a function of the applied uniaxial strain under different orientation angles (2°, 22°, 40°, 62°, 82°, and 90°). A linear fit is used to extract the gauge factor. The shaded area around the dashed lines indicates the uncertainty in the linear fit and provides a measurement of the uncertainty of the extracted gauge factor. d) Angular dependence of the ZrSe_3_ A exciton gauge factor in polar coordinates. All the tests are carried out with the strain axis parallel to the horizontal axis of the microscope and by keeping the incident linearly polarized light and flake *b*‐axis direction parallel to each other along the whole experiment to maximize the excitons intensities.

**Figure** **4**a shows a selection of differential reflectance spectra acquired while applying different uniaxial strain values almost parallel to the *b*‐axis (θ = 2°). Figure 4b shows other differential reflectance spectra versus strain acquired when the uniaxial strain is orthogonal to the *b*‐axis (θ = 90°). Note that a smooth polynomial background has been subtracted from the spectra to facilitate identifying the exciton peaks (see Figure S3 in the Supporting Information that compares some spectra before and after removing the background). The spectra have been fitted to a sum of two Gaussian peaks to accurately determine the energy of the A and B excitonic features. Figure 4c summarizes the A exciton peak energy versus uniaxial strain acquired for different alignment between the uniaxial straining direction and the ZrSe_3_ *b*‐axis. The strain gauge factor, defined as the spectral shift per % of uniaxial strain, strongly depends on the angle between the uniaxial straining direction and the *b*‐axis. It ranges from ≈95 meV %^−1^ for parallel configuration (θ = 2°) to ≈15 meV %^−1^ for the orthogonal configuration (θ = 90°). Figure 4d further summarizes the gauge factors determined for different alignment between the uniaxial strain direction and the ZrSe_3_ *b*‐axis.

The B exciton peak is much lower in intensity and it is wider, making the Gaussian fit less accurate. This motivated us to focus on the A exciton, that contains information about the lower energy direct band‐to‐band transition. Nonetheless, we address the reader to Figure S5 in the Supporting Information for a dataset where both A and B exciton energies have been determined as a function of strain. Figure S5 in the Supporting Information also shows the effect of compressive uniaxial strain (using a four‐points bending setup, see Scheme S1, Supporting Information) on the differential reflectance of a ZrSe_3_ flake, showing how the A and B exciton peaks redshift upon compression at a rate of ≈−80 meV %^−1^.

It is worth noting that the ZrSe_3_ gauge factor for uniaxial strain along the *b*‐axis is substantially larger than that of transition metal dichalcogenides (typically in the 30–60 meV %^−1^)^[^ ^15^ ^]^ and reproducible (see Figure S6, Supporting Information). In fact, this value is very close to that of InSe^[^ ^48^, ^49^ ^]^ and black phosphorus,^[^ ^29^ ^]^ the 2D materials with the largest strain gauge factors reported so far. However, both InSe and black phosphorus are sensitive to the environmental exposure and tend to degrade. We have found that the observed exciton strain tunability remains stable even after 4 months of exposure to atmospheric conditions (see Figures S7 to S10, Supporting Information).

We have measured a total of 11 ZrSe_3_ flakes obtaining comparable results (see Figures S11 to S19 of the Supporting Information). This illustrates the robustness of the observed anisotropy in the strain‐tunable A exciton energy. In order to get an insight about the maximum strain that the ZrSe_3_ flakes can sustain, Flakes 7, 8, and 9 were subjected to uniaxial strain until fracture (see Figures S16–S18 in the Supporting Information), revealing that the flake breakdown occurs between ≈0.8% and 1.2%.

To quantify the observed anisotropy in the strain tunability of the excitons, we define the anisotropy ratio of the gauge factor as

(2) $$Anisotropy\;ratio=\left({\text{GF}}_{\max}-{\text{GF}}_{\min}\right)/\left({\text{GF}}_{\max}+{\text{GF}}_{\min}\right)\cdot 100\%$$

where GF_min_ and GF_max_ stands for the minimum and maximum gauge factors, respectively. This formula gives 0% for perfectly isotropic material. For the studied ZrSe_3_ flakes, we get values ranging from 72% to ≈100%. Black phosphorus, another 2D material with a remarkable in‐plane structural anisotropy, showed only an ≈3% anisotropy ratio of the gauge factor.^[^ ^29^ ^]^ **Table** **1** compares the gauge factors along different crystal directions for ZrSe_3_ and those reported for black phosphorus in ref. [29]. Table 1 also includes information about the thickness of the ZrSe_3_ flakes. Samples 1, 2, 6, 8, and 11 were characterized by atomic force microscopy (AFM). For the other ZrSe_3_ samples, an estimation of their thickness has been obtained from the optical contrast extracted from the red channel of the optical images (see Figure S20 in the Supporting Information for details). We have not found any clear thickness dependence in the resulting gauge factor nor anisotropy ratio. We attribute this to the fact that the thinner studied flake was ≈8 nm (approximately eight layers) that is expected to show bulk‐like properties (see Figure S21, Supporting Information). At the present, we could not exfoliate thinner flakes with a large enough area to study strain engineering experiments with differential reflectance. Note that for a good strain transfer, flakes of at least 10 × 10 µm^2^ are selected.

**Table 1:** Summary of the reported exciton shift upon uniaxial strain along different in‐plane directions for ZrSe_3_ and black phosphorus

Reference	Material	Thickness [nm]	Gauge factor [meV %^−1^]		Anisotropy ratio [%]
			Axis 1	Axis 2	
This work	ZrSe_3_ (Sample 1, Figure 3)	16.0 ^AFM^	+ (15.1 ± 2.4)	+ (95.7 ± 4.1)	72.7
	ZrSe_3_ (Sample 2, Figure S6, Supporting Information)	14.5 ^AFM^	≈0	+ (75.7 ± 5.3)	≈100
	ZrSe_3_ (Sample 3, Figure S7, Supporting Information)	≈8 ^OPT^	+ (5.7 ± 3.2)	+ (70.2 ± 0.6)	84.9
	ZrSe_3_ (Sample 4, Figure S8, Supporting Information)	≈16 ^OPT^	+ (4.4 ± 3.9)	+ (91.7 ± 8.9)	90.8
	ZrSe_3_ (Sample 5, Figure S9, Supporting Information)	≈16 ^OPT^	+ (2.0 ± 2.8)	+ (82.7 ± 3.9)	95.3
	ZrSe_3_ (Sample 6, Figure S10, Supporting Information)	8.3 ^AFM^	+ (4.6 ± 3.0)	+ (92.0 ± 5.7)	90.5
	ZrSe_3_ (Sample 7, Figure S11, Supporting Information)	≈16 ^OPT^	–	+ (70.1±4.0)	–
	ZrSe_3_ (Sample 8, Figure S12, Supporting Information)	12.8 ^AFM^	–	+ (64.1 ± 3.4)	–
	ZrSe_3_ (Sample 8, Figure S12, Supporting Information)^a)^	12.8 ^AFM^	+ (10.5 ± 4.1)	+ (77.6 ± 3.6)	76.2
	ZrSe_3_ (Sample 9, Figure S13, Supporting Information)	≈13 ^OPT^	–	+ (74.3 ± 5.0)	–
	ZrSe_3_ (Sample 10, Figure S14, Supporting Information)^b)^	≈15 ^OPT^	–	+ (68.7 ± 7.9) to + (81.2 ± 4.7)	–
	ZrSe_3_ (Sample 10, Figure S5, Supporting Information)^c)^	≈15 ^OPT^	–	− (78.3 ± 4.0)	–
	ZrSe_3_ (Sample 11, Figure S5, Supporting Information)^d)^	14.2 ^AFM^	+ (9.6 ± 5.0) to + (22.6 ± 5.0)	+ (83.8 ± 2.0) to + (93.0 ± 5.5)	57.5 to 81.3
	ZrSe_3_ (ab initio)	Bulk	+ 10.9	+ 58.7	68.7
^[^ ^29^ ^]^	BP	3.3	+ 117	+ 124	2.9

^a)^ Measurement carried out 4 months after the sample fabrication (sample stored in air)

^b)^ Measurement acquired in five straining/releasing cycles

^c)^ Measurement acquired by applying compressive strain

^d)^ Measurements carried out at different times along 1 month of exposure to air.

**Figure 5:**
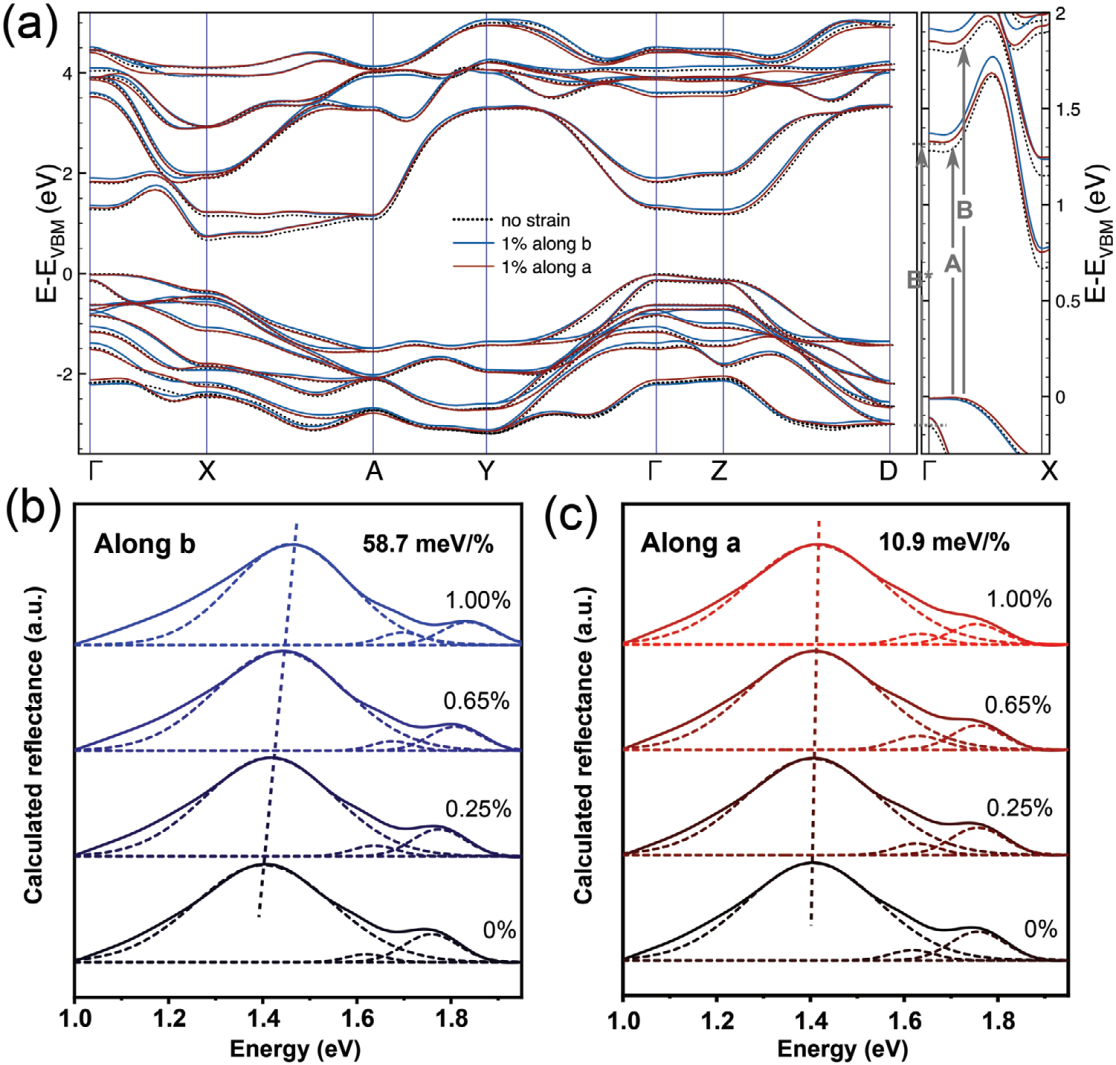
Calculated band structure and reflectance spectra for uniaxial strains along the *b‐* and *a*‐axis. a) Band structure, calculated within the GW approximation, for different straining situations: 0% strain (dotted black), +1% uniaxial strain along *a*‐axis (red), and +1% uniaxial strain along *b*‐axis (blue). We have selected the path in the first Brillouin zone: Γ = (0,0,0), *X* = (½,0,0), *A* = (½,½,0), *Y* = (0,½,0), *Z* = (0,0,½), *D* = (0,½,½). A zoomed‐in region in the Γ–*X* has been plotted to highlight the direct band‐to‐band transitions that originate the A, B, and B* excitons. b,c) Calculated reflectance spectra (solid lines) for different uniaxial tensile strains along the *b*‐axis (blue) and *a*‐axis (red), respectively. The dashed lines represent a fit with three Gaussian functions to determine the position of the A, B, and B* exciton peaks.

We have also carried out ab initio calculations to investigate further this strongly anisotropic response to strain of ZrSe_3_. **Figure** **5**a shows the electronic band structure of bulk ZrSe_3_ as calculated within the GW approximation for the unstrained structure and for 1% strain applied along the *a* and *b* axes. For the unstrained structure, we obtain a direct electronic band‐gap of 1.29 eV at the Γ point, and an indirect band gap of 0.66 eV between Γ and X points. The GW approximation corrects the density functional theory (DFT) band gaps (direct, 0.46 eV, indirect 0.15 eV) bringing them closer to the experimental values. The main effect of the GW approximation is a rigid upward shift of the conduction bands in the whole Brillouin zone. We clearly see that a 1% tensile strain along the *b*‐axis shifts the direct band gap up by about +90 meV while the same strain along the *a*‐axis only shifts the bands by +45 meV. Interestingly, the anisotropy in the strain‐tunable indirect band gap is less pronounced, +100 meV %^−1^ along the *b*‐axis and +85 meV %^−1^ along the *a*‐axis, in good agreement with previously reported DFT calculations that only focused on the indirect band‐gap strain tunability without considering excitonic effects.^[^ ^50^ ^]^

In order to directly compare with our experimental findings, we have calculated reflectance spectra (Figure 5b,c) via solving the Bethe–Salpeter's equation (see the Experimental Section for further information). The spectra present two prominent peaks around ≈1.4 and ≈1.75 eV, due to the generation of A and B excitons respectively. These two peaks are lower in energy than the experimental values, but their positions are consistent with the smaller band gap we obtained in the GW approximation. Interestingly, our calculation also predicts the presence of another exciton, that we label as B*, with an energy between that of A and B excitons but with a very low intensity. Figure 5a includes a zoomed‐in plot of the band structure, indicating the different band‐to‐band transitions that originate the A, B, and B* excitons.

We calculated the effect of tensile strain along the *a‐* and *b*‐axis, considering incident light polarized along the *b‐*axis of ZrSe_3_. For the strain along the *b*‐axis, we clearly observe a blueshift of the exciton peaks (at a rate of ≈59 meV %^−1^ of uniaxial strain for the A exciton), consistent with an increase in the electronic band gap. The exciton blueshift is much less pronounced (only ≈11 meV %^−1^ for the A exciton) when uniaxial strain is applied along the *a*‐axis, reflecting the minimal effect of strain applied along that crystal direction on the band structure. In order to get an insight about the physical origin of the anisotropy in the strain‐tunable exciton energies, we have analyzed the contribution of the different atoms, and their orbitals, to the band structure (see Figures S22 to S24 in the Supporting Information and related discussion). Our analysis allows us to conclude that the conduction bands, around Γ, are mainly determined by the Zr d*_xy_* and d*_yz_* orbitals. This fact suggests that deforming the unit cell along *y* (*b*‐axis) might have a more significant impact since it modifies the bonds containing the d*_xy_* and d*_yz_* orbitals (see Figure S25 in the Supporting Information). At the same time, a deformation along *x* (*a*‐axis) only affects the d*_xz_* orbital. Regarding the valence band maximum around the Γ point, our analysis concludes that it is mainly determined by the p*_z_* orbital of Se_2_ atoms. This observation explains rather the insensitiveness of the valence band maximum to strain applied to both the *a* and *b* directions (parallel to *x* and *y* in our case) as pointed out by our band structure calculations.

Note that the theoretical anisotropy ratio is ≈69%, slightly lower than the experimental values that span from 72% to nearly 100%. We attribute this underestimation of the anisotropy to the fact that we neglected the Poisson's effect in our calculation as we could not find any previously peer‐reviewed reported value of the Poisson's ratio of ZrSe_3_. According to the Poisson's effect, a tensile strain along the *a*‐axis will be accompanied by a small compression along the *b*‐axis that could effectively reduce the exciton strain‐induced shift. In fact, a compression along *b*‐axis will lead to a redshift of the excitons (see Figure S5 of the Supporting Information) which could partially compensate the blueshift produced by the tension along the *a*‐axis, thus increasing the anisotropy ratio.

## 4 Conclusions

In summary, we subjected thin ZrSe_3_ flakes to uniaxial strain along different crystal orientations. We found that the reflectance spectra showed a peak, associated with a direct band gap transition, that blueshifts upon uniaxial tension, indicating a tension‐induced band gap increase. Interestingly, the spectra shift rate strongly depends on the direction along which the strain is being applied. While strain along the *b*‐axis yielded a spectral shift up to ≈60–95 meV %^−1^, strain applied along the *a*‐axis only yielded a shift of ≈0–15 meV %^−1^. Ab initio calculations further verified the large anisotropic strain‐tunable band‐gap transitions observed in our experiments.

## 5 Experimental Section

### Sample Fabrication

Bulk ZrSe_3_ crystals (from HQ Graphene) were exfoliated with Nitto SPV 224 tape and transferred to a Gel‐Film (Gel‐Pak WF × 4 6.0 mil) substrate. Then, the surface of the Gel‐Film substrate was scanned under an optical microscope operated in transmission mode to identify the flakes. Once the desired flake was identified, it could be transferred onto a desired location of a target substrate by means of an all‐dry deterministic placement method.^[^ ^41^, ^42^, ^43^ ^]^

### Strain‐Dependent Differential Reflectance Spectroscopy

The disk‐shaped flexible substrate containing the desired flake on its center was loaded into the three‐points bending system and the whole system was mounted under the objective of an optical microscope (Motic BA310 Met‐T) system supplemented with a homebuilt micro‐reflectance module based on a fiber‐coupled CCD spectrometer (CCS200/M, Thorlabs) as previously reported.^[^ ^46^ ^]^ The assigned flake was centered with respect to the central pivot using microscope inspection. Importantly, differential reflectance was used instead of photoluminescence, commonly employed to monitor the effect of strain on the optical properties of 2D semiconductors, given that photoluminescence measurements led to a laser‐induced burning of thin ZrSe_3_ flakes.

### Ab Initio Calculations

The electronic band structure and the optical reflectance were calculated through the GW approximation starting from DFT calculations. The atomic structure was initially relaxed, until the residual force between the atoms was below 4.0 × 10^−4^ eV Å^−1^. The lattice parameters, *a* = 5.44 Å, *b* = 3.73 Å, and *c* = 9.45 Å with an angle between *a* and *c* of 97.6° were determined. All calculations were performed with scalar relativistic generalized gradient approximation‐Perdew–Burke–Ernzerhof pseudopotentials in the optimized norm‐conserving Vanderbilt set.^[^ ^51^, ^52^ ^]^ The Quantum Espresso suite was used for the calculations.^[^ ^53^, ^54^ ^]^ An energy cut‐off of 10^3^ eV and a uniform mesh of k‐points of 6 × 6 × 6 in the Brillouin zone were set. The GW calculations were performed using the Yambo code^[^ ^55^, ^56^ ^]^ starting from the Quantum Espresso output. The optical spectra were obtained by solving the Bethe–Salpeter equation starting from the GW calculation in the plasmon‐pole approximation. This calculation provided the real and imaginary part of the dielectric function of the material. From there, the reflectance was evaluated considering that the light was perpendicular to the sample. The line width of the spectra was therefore obtained from the solution of that equation without any further approximation. In particular, the method was able to evaluate the electronic self‐energy thus providing the line width. After modifying the unit cell parameters to take into account the uniaxial strain, the atomic position was optimized till the residual force was below 4.0 × 10^−4^ eV Å^−1^.

### Scanning Transmission Electron Microscopy (STEM)

For STEM characterization, ZrSe_3_ flakes were mechanically transferred onto a holey Si_3_N_4_ membrane and characterized using an aberration‐corrected JEOL JEM‐ARM 200cF electron microscope equipped with a cold field emission gun and operated at 80 kV.

## Conflict of Interest

The authors declare no conflict of interest.

## Supporting information

Supporting Information

## Data Availability

Research data are not shared.
