# Supplementary material for: Strongly Anisotropic Strain‐Tunability of Excitons in Exfoliated ZrSe3
Source: Adv Mater. 2021 Oct 15;34(1):2103571. doi: 10.1002/adma.202103571 (PMC11468225; doi:10.1002/adma.202103571)
Supplement: Supplementary file 1 — Supporting Information [file ADMA-34-2103571-s001.pdf]

ADVANCED MATERIALS

Supporting Information

for *Adv. Mater.,* DOI: 10.1002/adma.202103571

Strongly Anisotropic Strain-Tunability of Excitons in Exfoliated $ZrSe_3$

Hao Li, Gabriel Sanchez-Santolino, Sergio Puebla, Riccardo Frisenda, Abdullah M. Al-Enizi, Ayman Nafady, Roberto D'Agosta, and Andres Castellanos-Gomez**

Supporting Information

Strongly anisotropic strain-tunability of excitons in exfoliated $ZrSe_3$

Hao Li, Gabriel Sanchez-Santolino, Sergio Puebla, Riccardo Frisenda, Abdullah M. Al-Enizi, Ayman Nafady, Roberto D'Agosta, Andres Castellanos-Gomez**

Atomic force microscopy characterization of the $ZrSe_3$ flake measured in the main text (sample 1)
Optical images of a $ZrSe_3$ flake before and after Raman and photoluminescence testing
Baseline subtraction in the differential reflectance spectra
Differential reflectance spectra using non-polarized incident light
Effect of uniaxial compression on the reflectance spectra and strain tunable B exciton
Four-points bending configuration to apply compressive strain
Reproducibility test
Angle-resolved micro-reflectance spectra of a $ZrSe_3$ sample (Sample 8), under different uniaxial strain from 0%-0.65%, after 4 months of air exposure
Comparison between the strain tunable exciton energy in Sample 8 right after sample fabrication and after 4 months of storage in air
Optical images of Sample 11 taken at different days after its fabrication
Comparison between the strain tunable exciton energy (for strains parallel and perpendicular to b-axis) in Sample 11 acquired at different days after sample fabrication
Study of the anisotropic strain tunable reflectance spectra on samples 2, 3, 4, 5 and 6
Study of the maximum achievable strain before rupture, samples 7, 8 and 9
Study of the reproducibility of the strain tunable reflectance spectra, sample 10
Relationship between the optical contrast, extracted from the red channel of the optical images of the $ZrSe_3$ flakes, with the thickness measured with AFM
The density of states (DOS) versus the electron energy for the monolayer, 5-layer and bulk $ZrSe_3$
The band structure of ZrSe3 split into the atomic contributions.

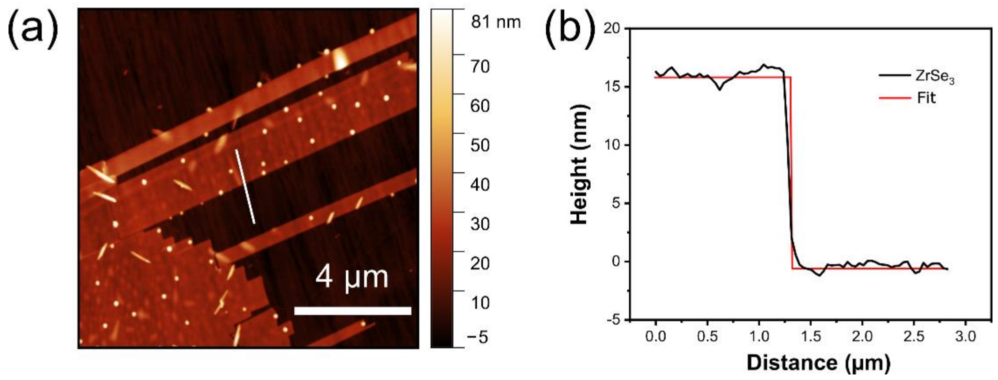

Figure S1. Atomic force microscopy characterization of the $ZrSe_3$ flake measured in the main text (sample 1). (a) AFM topography image. (b) Line profile measured along the white line in (a) to show the thickness of the flake.

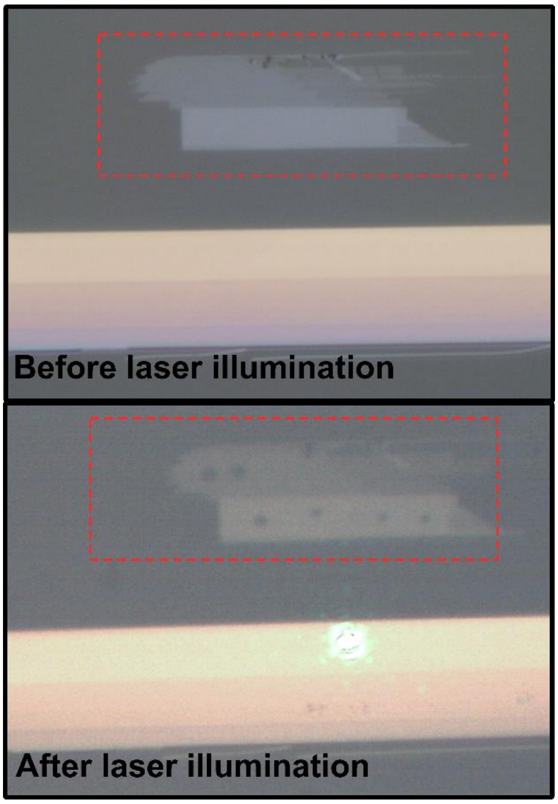

Figure S2. Optical images of a $ZrSe_3$ flake before and after Raman and photoluminescence testing. We could not observe any Raman or PL spectra because of the laser-induced damage. Wavelength: 532 nm, objective: 50×. Acquisition time: 20 s, power: 0.129 mW, spot size: 2 μm, power density: $4.1 \cdot 10^{-5}$ W/μm^2.

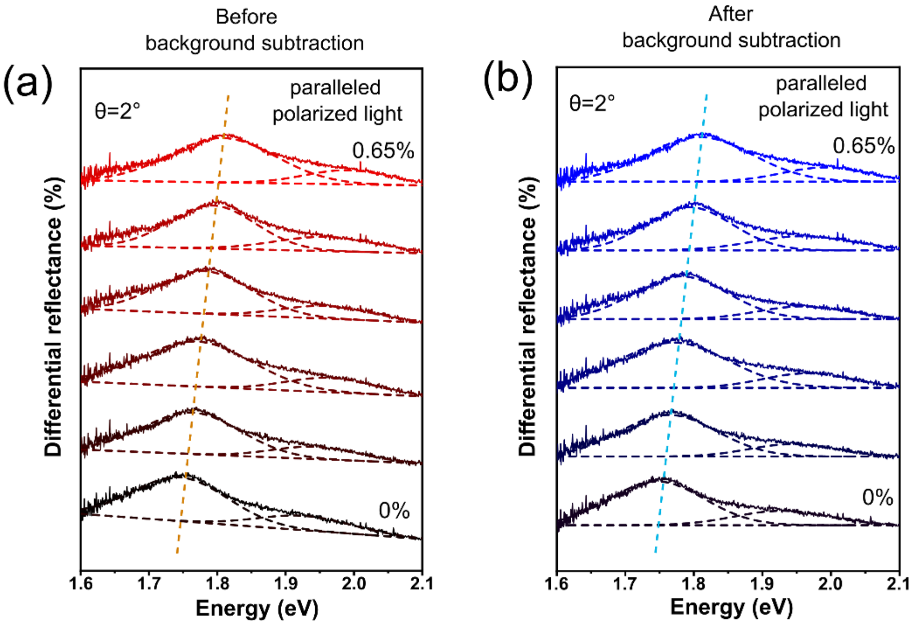

Figure S3. Baseline subtraction in the differential reflectance spectra. (a) Differential reflectance spectra acquired (sample 1) for different uniaxial strains applied along the *b*-axis before subtracting the background. (b) Same spectra after background subtraction.

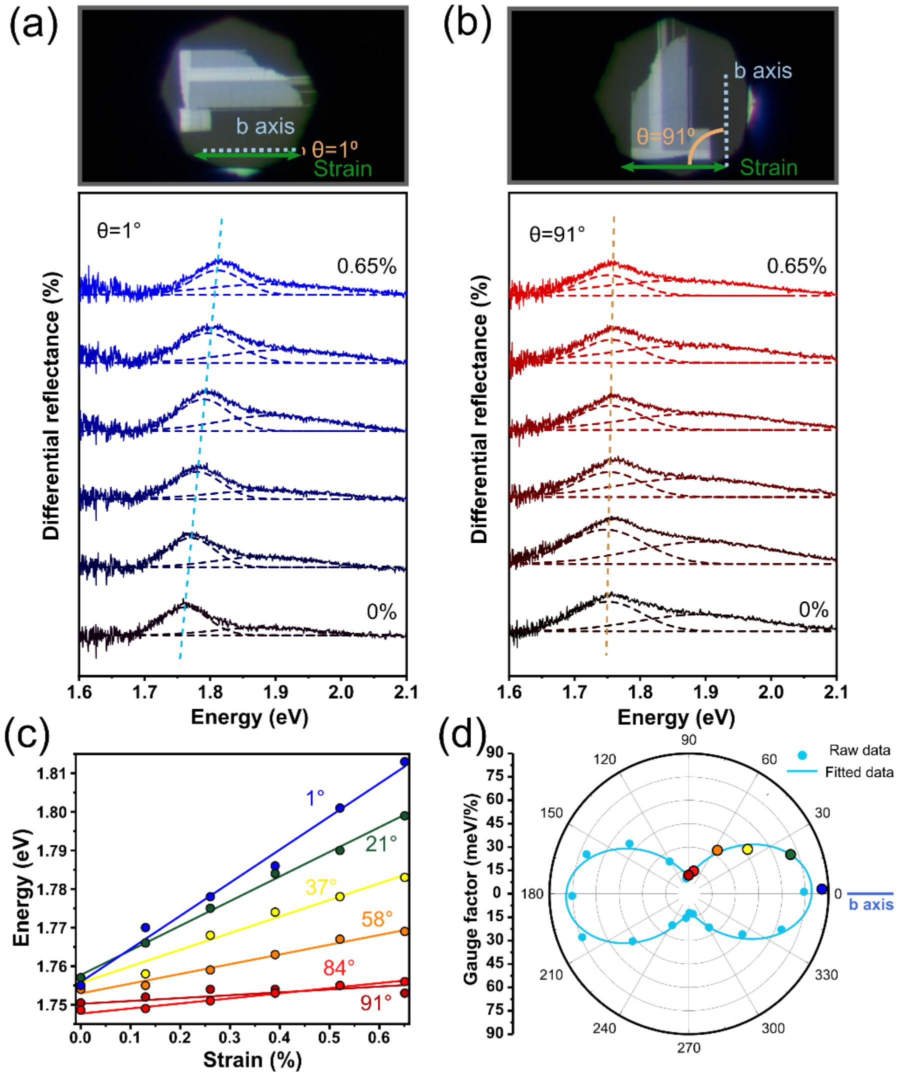

Figure S4. Differential reflectance spectra using non-polarized incident light. Same figure as Figure 4 of the main text (sample 1) but using non-polarized light instead of linearly polarized light along the *b*-axis. The exciton peaks are less pronounced than using linearly polarized light but the strain tunability is similar in magnitude and trend. (a, b) The micro-reflectance spectra acquired when the uniaxial strain direction is parallel to the *b*-axis and the *a*-axis of the $ZrSe_3$ flake, respectively. (c) The energy of the excitonic peak as a function of the applied uniaxial strain under different orientation angles (1º, 21º, 37º, 58º, 84º and 91º). A linear fit is utilized for extracting the gauge factor. (d) The experimental and fitted gauge factor change of the excitonic peak of $ZrSe_3$ in polar coordinates.

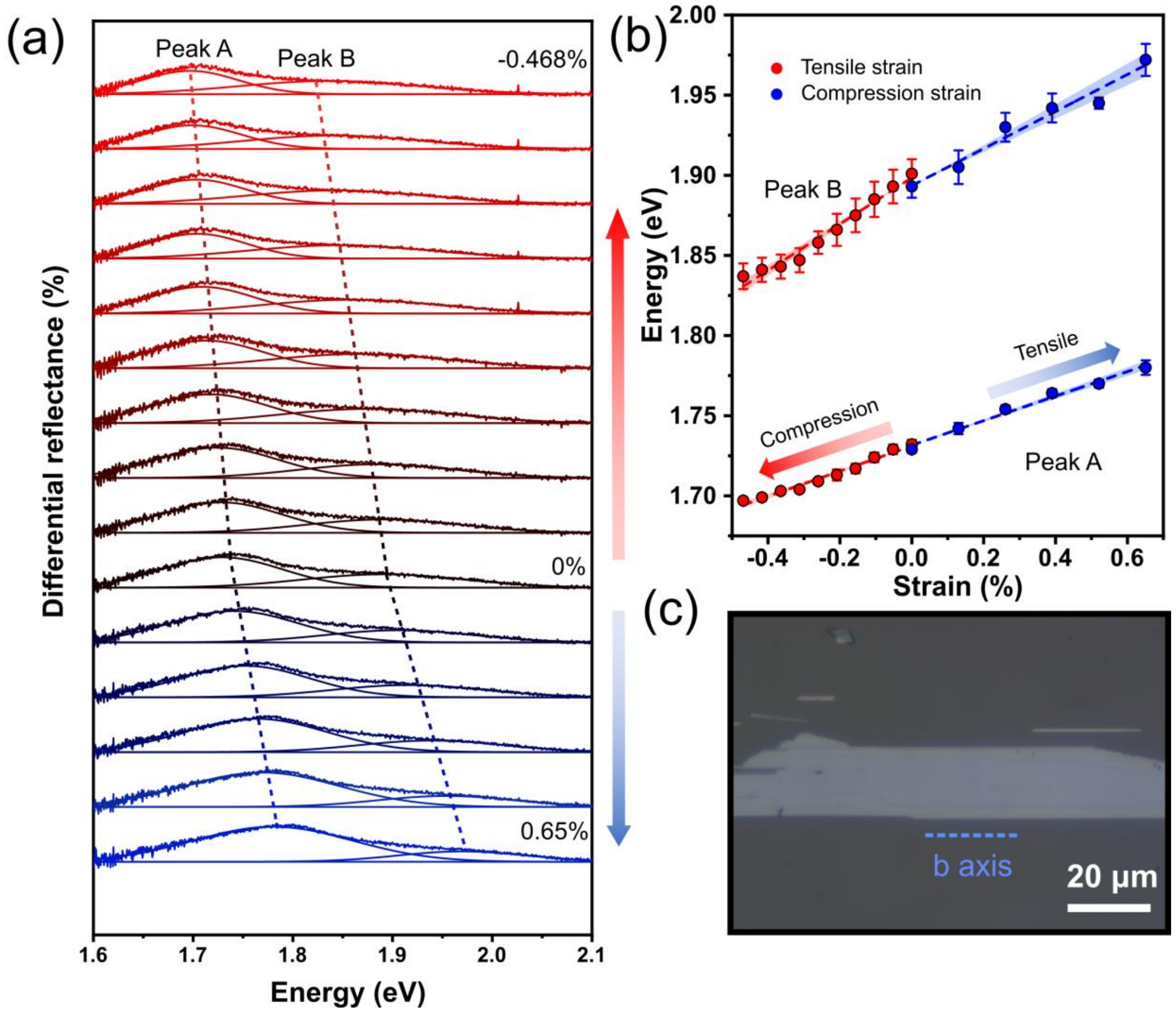

Figure S5. Effect of uniaxial compression on the reflectance spectra and strain tunable B exciton. (a) Differential reflectance spectra acquired on Sample 10 for different uniaxial strains along b ranging from -0.468% (compression) to +0.65% (tension). (b) Energy of the A and B exciton peaks as a function of strain. (c) Optical microscopy image of the sample 10. A four-points bending setup has been used for this measurement (see Scheme S1).

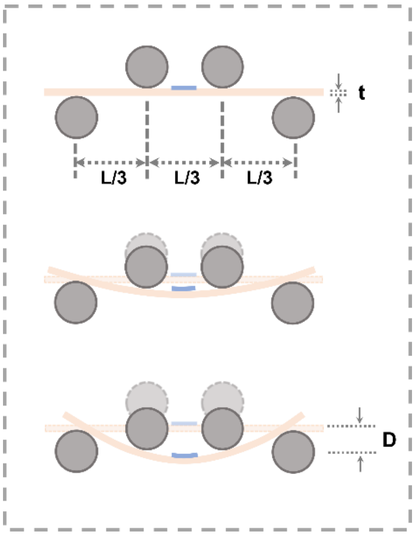

Scheme S1. Four-points bending configuration to apply compressive strain. The four-points bending setup calculation is based on reference [S1]: $\varepsilon=27*D*t/(5\cdot L^2)$. D represents the deflection of the plate. t represents the thickness of the plate, and L is the distance between the two outer cylinders (36 mm).

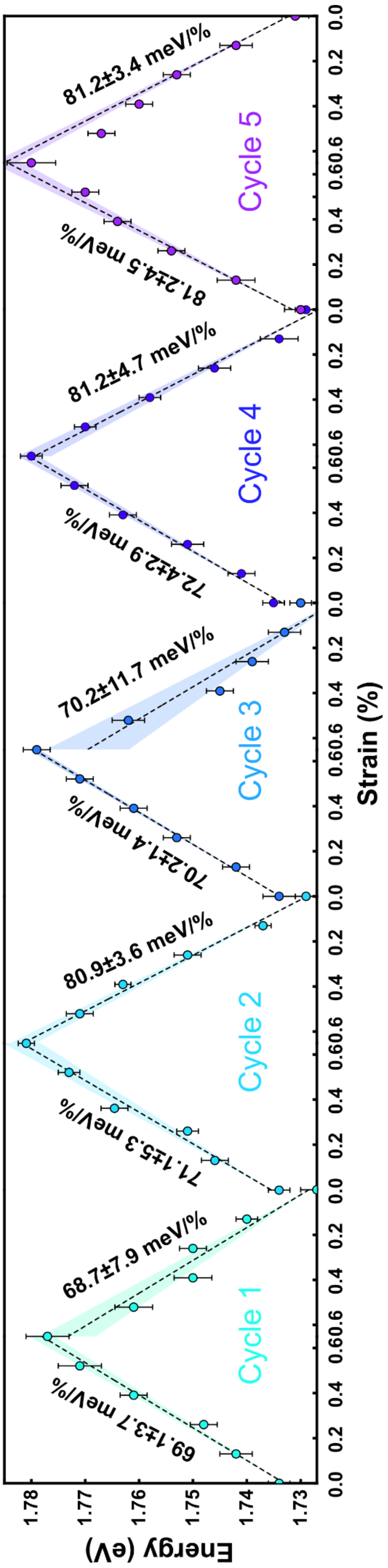

Figure S6. Reproducibility test. A exciton energy as a function of uniaxial tensile strain for 5 different cycles of strain loading/unloading. The measurements have been carried out in sample 10.

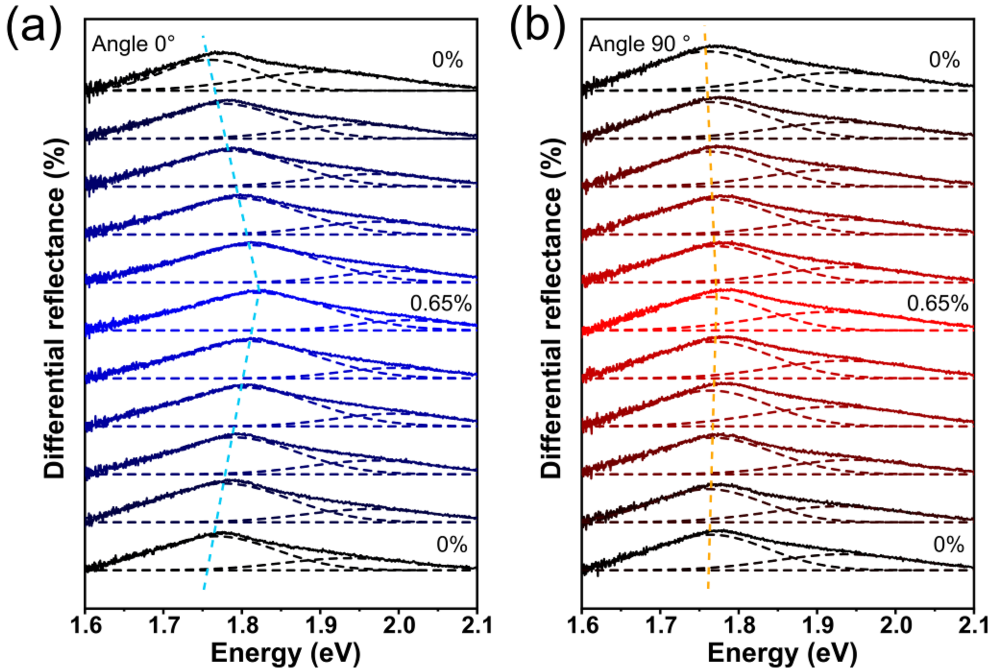

Figure S7. Angle-resolved micro-reflectance spectra of a $ZrSe_3$ sample (Sample 8), under different uniaxial strain from 0%-0.65%, after 4 months of air exposure. (a, b) Micro-reflectance spectra acquired when the uniaxial strain direction is parallel to the *b*-axis and the *a*-axis of the $ZrSe_3$ flake, respectively.

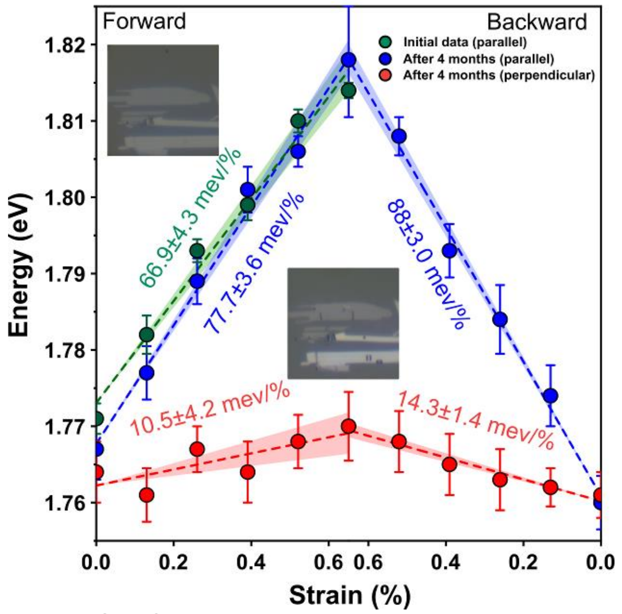

Figure S8. Comparison between the strain tunable exciton energy in Sample 8 right after sample fabrication and after 4 months of storage in air. (Inset, up) Optical image of the flake right after exfoliation and transfer. (Inset, lower) Optical image of the flake after 4 months in air.

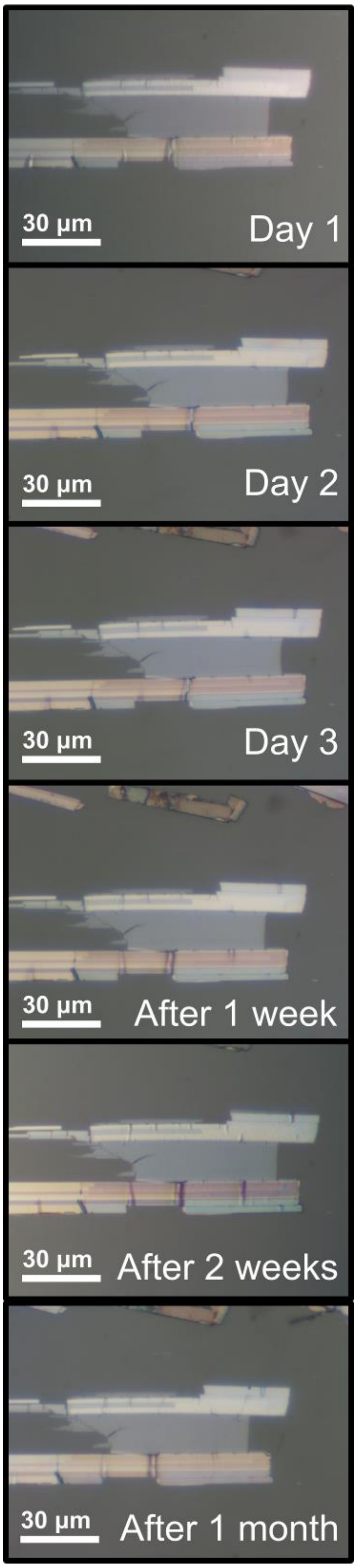

Figure S9. Optical images of Sample 11 taken at different days after its fabrication.

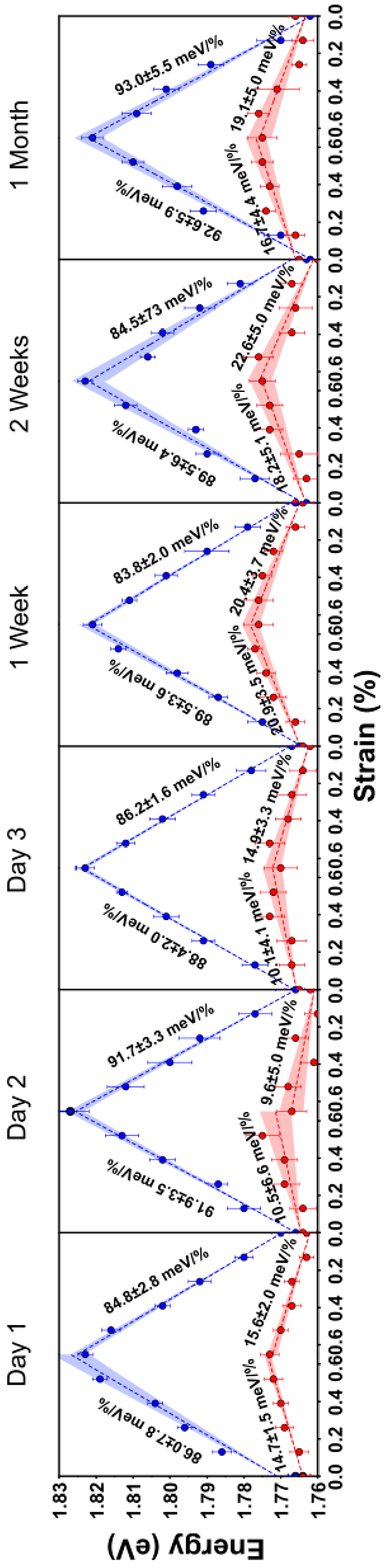

Figure S10. Comparison between the strain tunable exciton energy (for strains parallel and perpendicular to b-axis) in Sample 11 acquired at different days after sample fabrication.

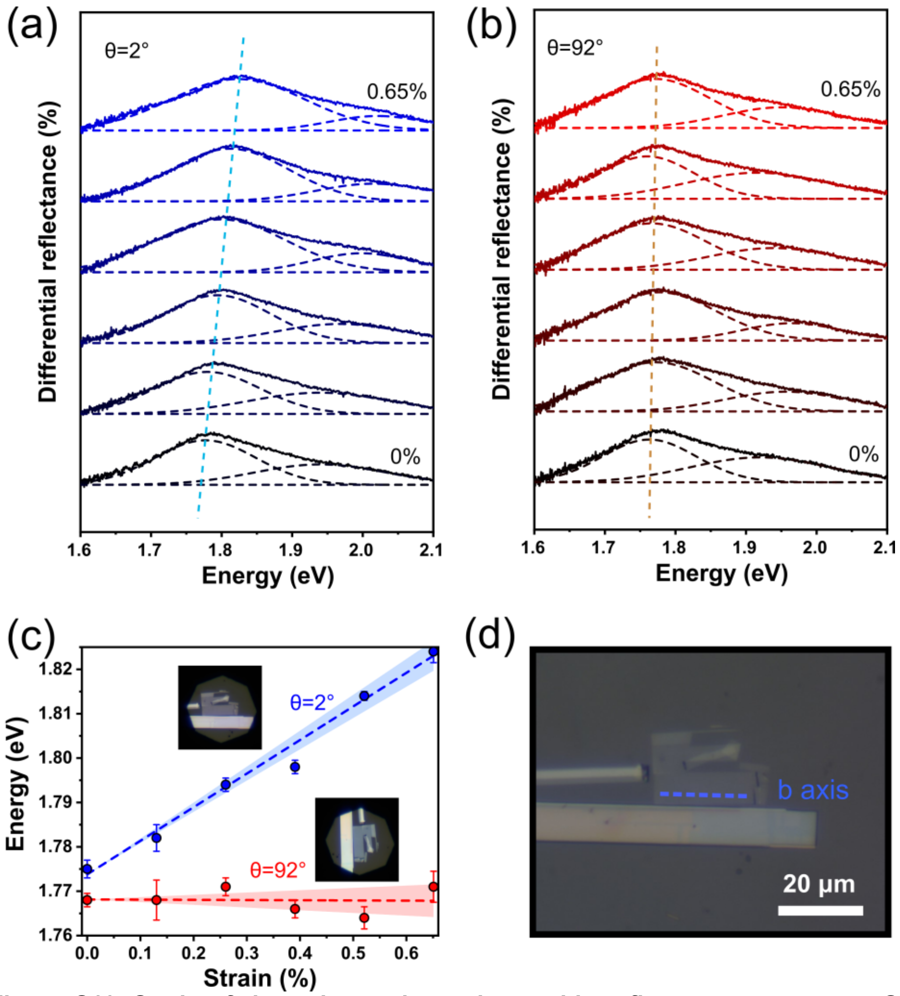

Figure S11. Study of the anisotropic strain tunable reflectance spectra on Sample 2. (a, b) The micro-reflectance spectra acquired when the uniaxial strain direction is parallel to the *b*-axis and the *a*-axis of the $ZrSe_3$ flake, respectively. (c) The energy of the A excitonic peak as a function of the applied uniaxial strain for the parallel and perpendicular to *b*-axis configurations. A linear fit is utilized for extracting the gauge factor. (d) Optical microscopy image of the sample 2.

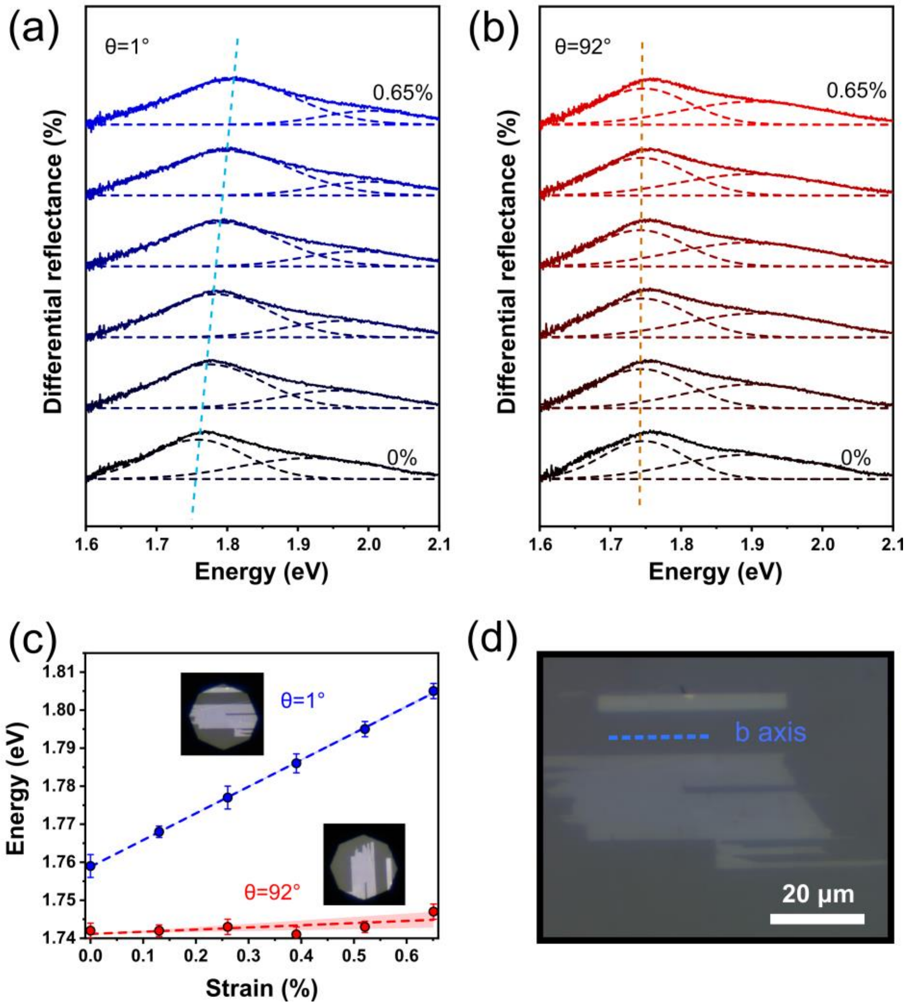

Figure S12. Study of the anisotropic strain tunable reflectance spectra on Sample 3. (a, b) The micro-reflectance spectra acquired when the uniaxial strain direction is parallel to the *b*-axis and the *a*-axis of the $ZrSe_3$ flake, respectively. (c) The energy of the A excitonic peak as a function of the applied uniaxial strain for the parallel and perpendicular to *b*-axis configurations. A linear fit is utilized for extracting the gauge factor. (d) Optical microscopy image of the sample 3.

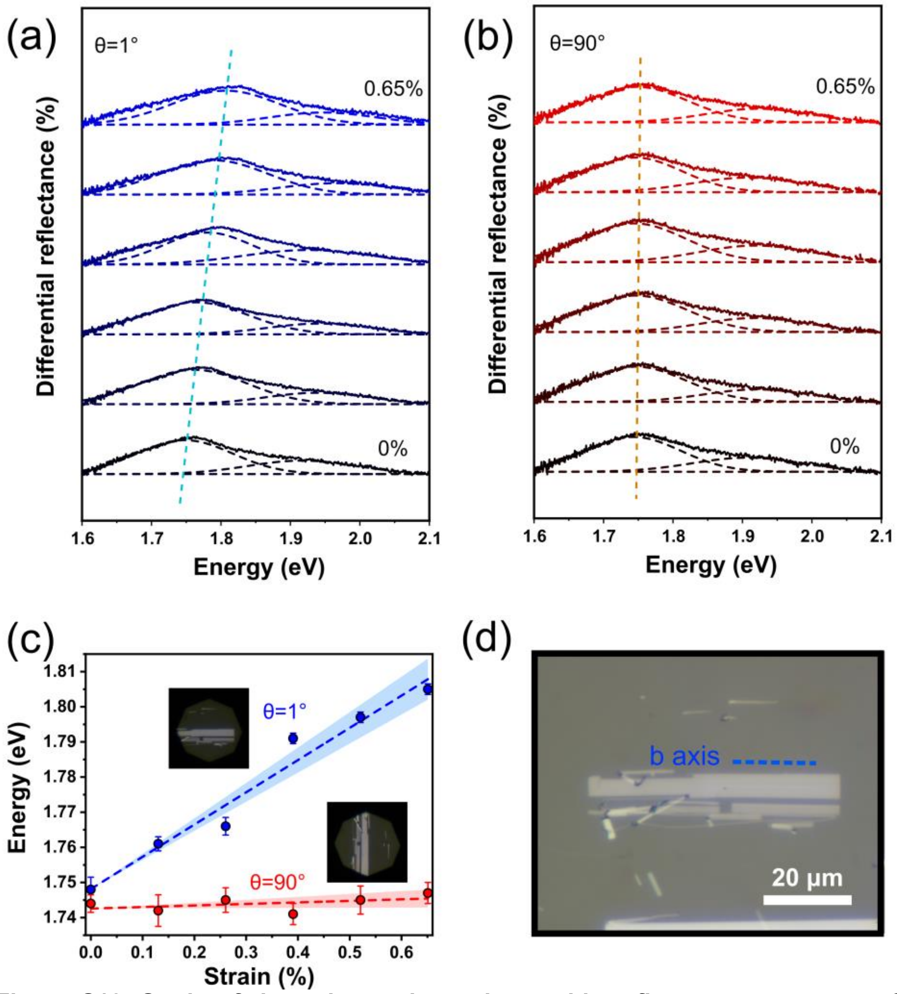

Figure S13. Study of the anisotropic strain tunable reflectance spectra on Sample 4. (a, b) The micro-reflectance spectra acquired when the uniaxial strain direction is parallel to the *b*-axis and the *a*-axis of the $ZrSe_3$ flake, respectively. (c) The energy of the A excitonic peak as a function of the applied uniaxial strain for the parallel and perpendicular to *b*-axis configurations. A linear fit is utilized for extracting the gauge factor. (d) Optical microscopy image of the sample 4.

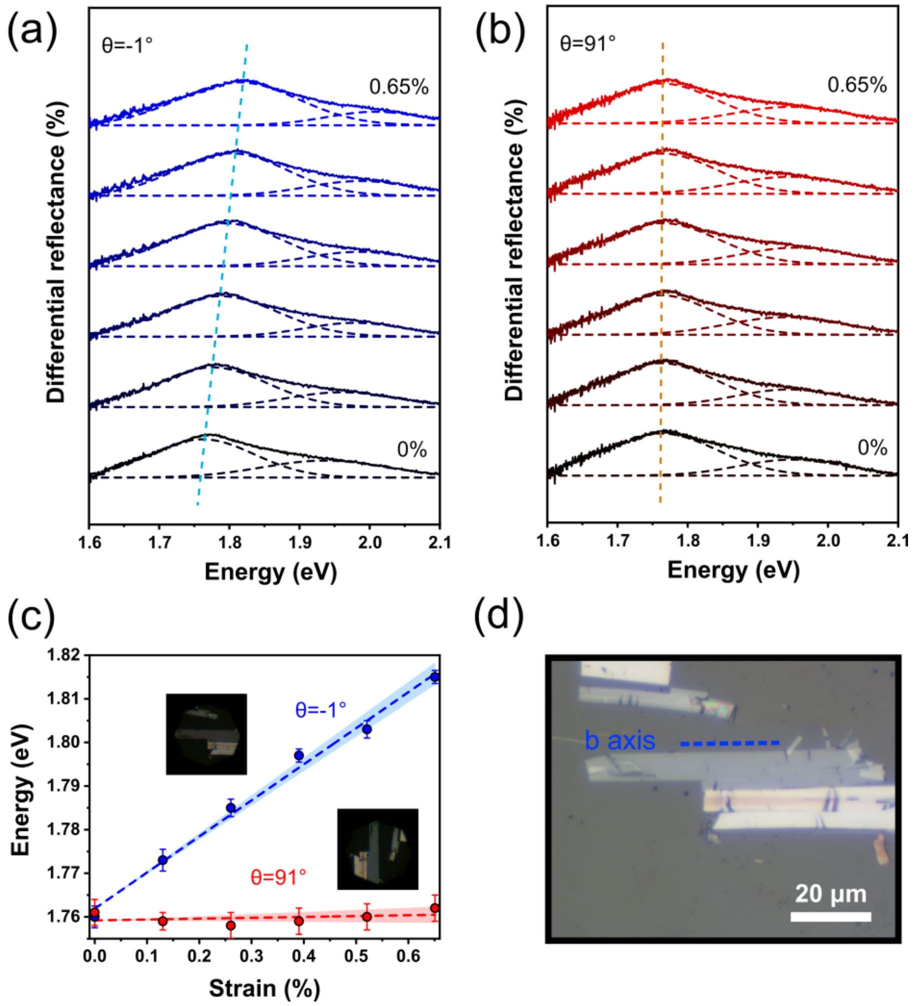

Figure S14. Study of the anisotropic strain tunable reflectance spectra on Sample 5. (a, b) The micro-reflectance spectra acquired when the uniaxial strain direction is parallel to the *b*-axis and the *a*-axis of the $ZrSe_3$ flake, respectively. (c) The energy of the A excitonic peak as a function of the applied uniaxial strain for the parallel and perpendicular to *b*-axis configurations. A linear fit is utilized for extracting the gauge factor. (d) Optical microscopy image of the sample 5.

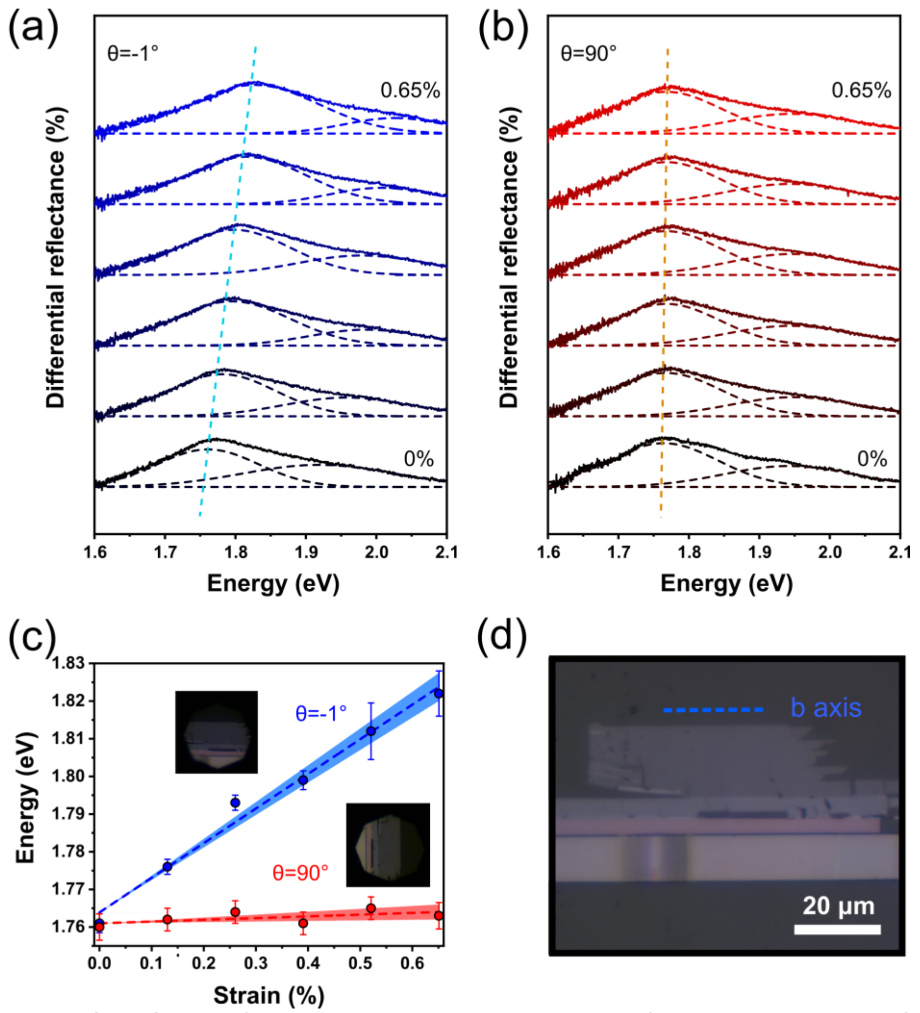

Figure S15. Study of the anisotropic strain tunable reflectance spectra on Sample 6. (a, b) The micro-reflectance spectra acquired when the uniaxial strain direction is parallel to the *b*-axis and the *a*-axis of the $ZrSe_3$ flake, respectively. (c) The energy of the A excitonic peak as a function of the applied uniaxial strain for the parallel and perpendicular to *b*-axis configurations. A linear fit is utilized for extracting the gauge factor. (d) Optical microscopy image of the sample 6.

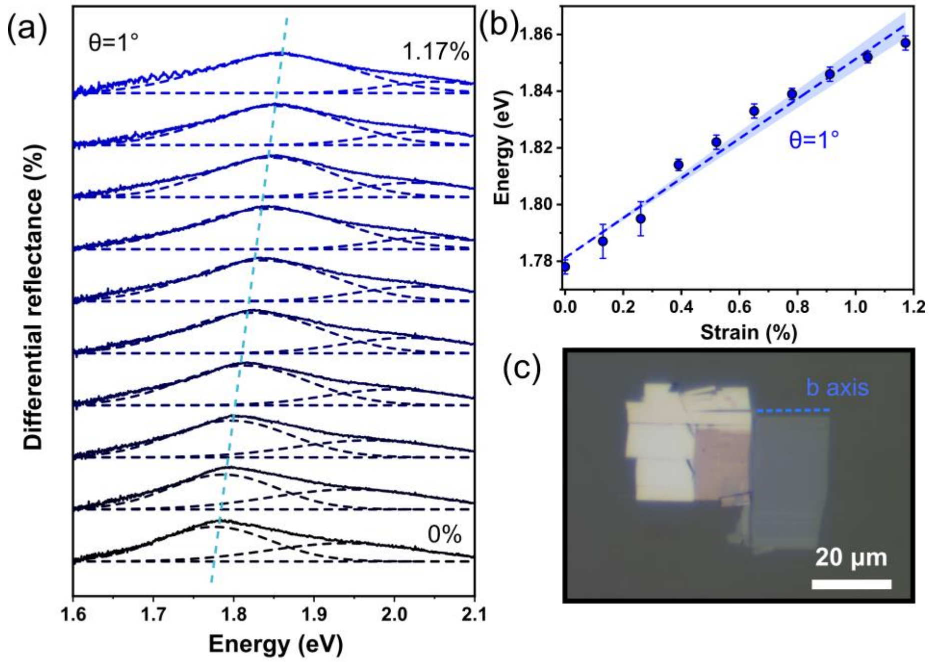

Figure S16. Study of the maximum achievable strain before rupture, Sample 7. (a) Micro-reflectance spectra acquired when the uniaxial strain direction is parallel to the *b*-axis of the $ZrSe_3$ flake. (b) The energy of the A excitonic peak as a function of the applied uniaxial strain for the parallel to *b*-axis configuration. (c) Optical microscopy image of the sample 7 before and after strain-induced rupture.

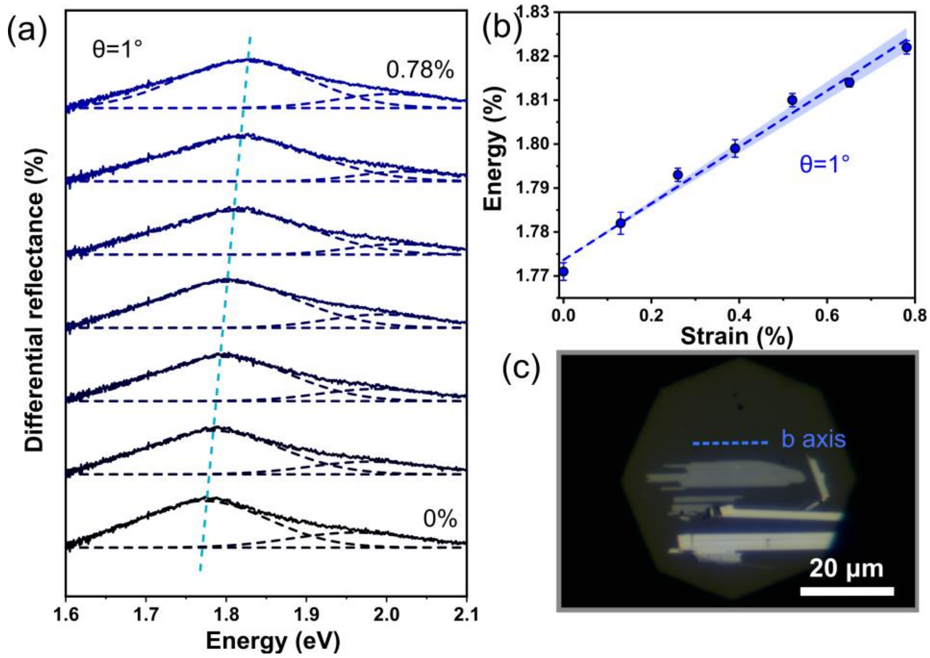

Figure S17. Study of the maximum achievable strain before rupture, Sample 8. (a) Micro-reflectance spectra acquired when the uniaxial strain direction is parallel to the *b*-axis of the $ZrSe_3$ flake. (b) The energy of the A excitonic peak as a function of the applied uniaxial strain for the parallel to *b*-axis configuration. (c) Optical microscopy image of the sample 8.

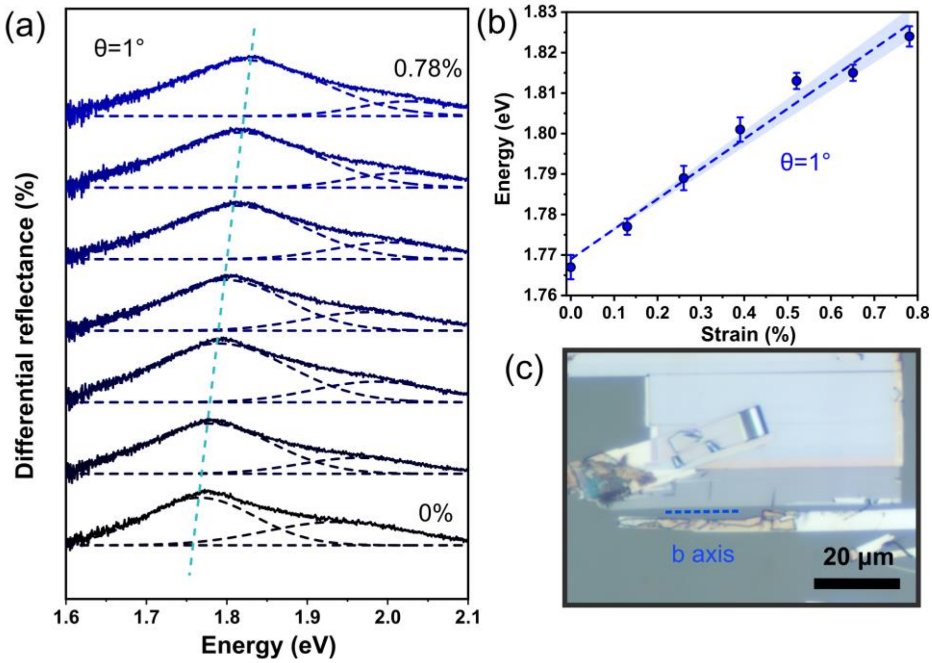

Figure S18. Study of the maximum achievable strain before rupture, Sample 9. (a) Micro-reflectance spectra acquired when the uniaxial strain direction is parallel to the *b*-axis of the $ZrSe_3$ flake. (b) The energy of the A excitonic peak as a function of the applied uniaxial strain for the parallel to *b*-axis configuration. (c) Optical microscopy image of the sample 9 before and after strain-induced rupture.

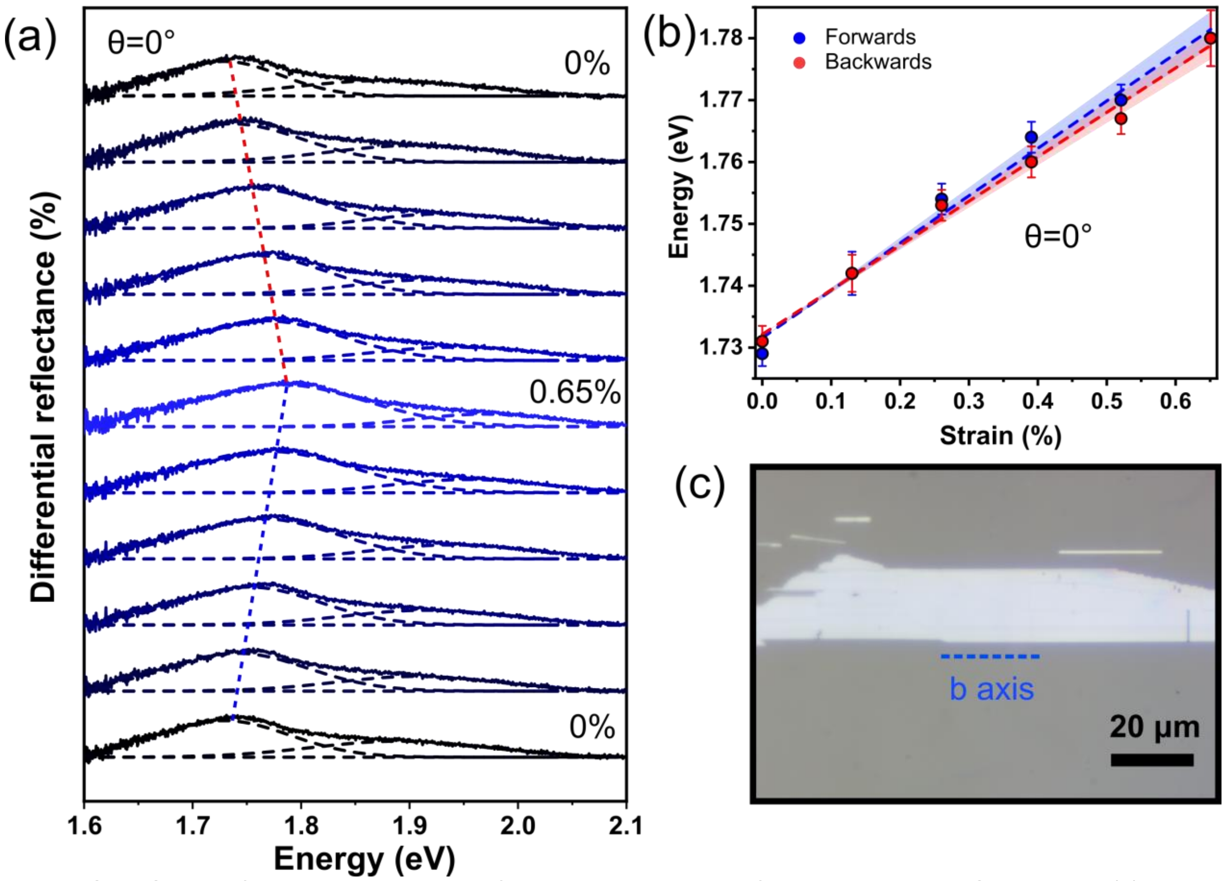

Figure S19. Study of the reproducibility of the strain tunable reflectance spectra, Sample 10. (a) Micro-reflectance spectra acquired when the uniaxial strain direction is parallel to the *b*-axis of the $ZrSe_3$ flake. (b) The

energy of the A excitonic peak as a function of the applied uniaxial strain for the parallel to *b*-axis configuration. (c) Optical microscopy image of the sample 10.

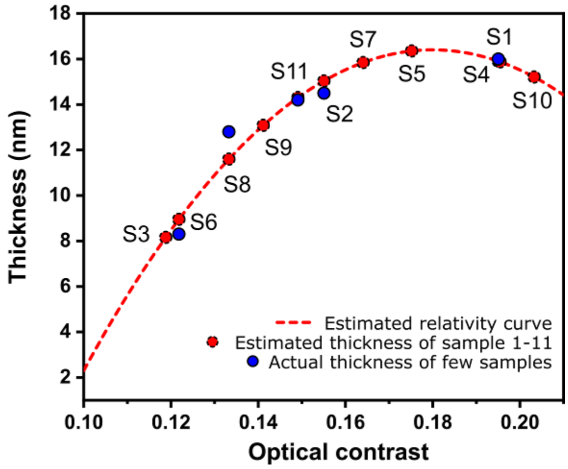

Figure S20. Relationship between the optical contrast, extracted from the red channel of the optical images of the $ZrSe_3$ flakes, with the thickness measured with AFM (blue dots). The experimental data has been fitted to a quadratic polynomial to estimate the thickness of the rest of the $ZrSe_3$ flakes from their optical contrast values.

Density of states (DOS): monolayer to bulk transition

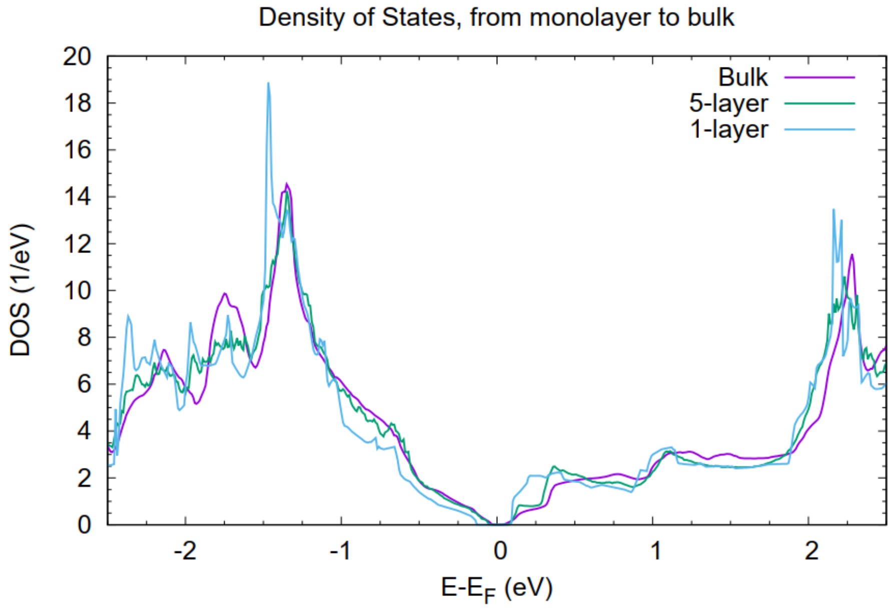

Figure S21. The density of states (DOS) versus the electron energy for the monolayer, 5-layer and bulk $ZrSe_3$.

In Figure S21, we report the density of states as a function of the energy for the monolayer, 5-layer and bulk $ZrSe_3$. Although there remain certain differences dictated mostly by the periodic nature of the bulk, the 5-layer and bulk DOS show similar features especially in the window -1 to 1 eV around the Fermi energy, namely the region of interest for optical applications.

Band structure analysis

To understand the anisotropic behaviour of the shift in the exciton peaks with applied strain, we have analysed the contribution to the band structure by the different atoms [S2]. Figure S22 reports the band structure projected on each of the atoms in the unit cell. The contribution is split on the Zr and two Se atoms. Indeed, not all the Se atoms are equivalent. Indeed, Se_2 here is the atom more internal and binds the two Zr atoms. Se_1 instead is one of the external atoms which binds with van der Walls forces the multilayer structure. From the figure, we see

that the valence band at the Γ point is dominated by the contribution of the Se_2 atom, while the Zr determines the conduction bands. Thus, we focus on those two atoms. (Notice that there are 4 Se atoms equivalent to Se_1, 2 Se atoms equivalent to Se_2, and two Zr atoms.)

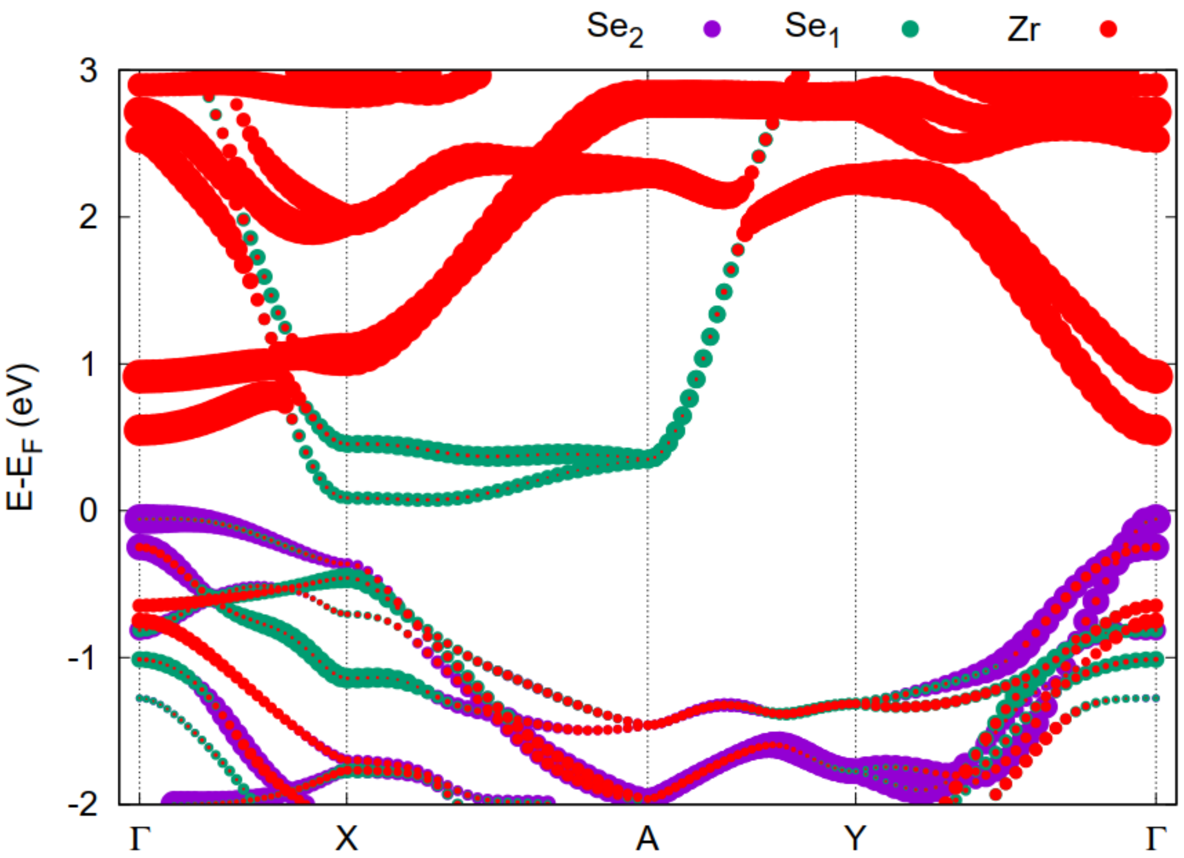

Figure S22: The band structure of $ZrSe_3$ split into the atomic contributions.

In Figure S23, we split the contribution of the Zr atoms in the contribution of its d states. Our analysis allows us to conclude that the conduction bands, around Γ, are mainly determined by the Zr d_{xy} and d_{yz} orbitals. The d_{xz} orbital has a contribution only at large energy. This fact suggests that deforming the unit cell along y (or b) might have a more significant impact since it modifies the bonds containing the d_{xy} and d_{yz} orbitals. At the same time, a deformation along x (or a) only affects the d_{xz} orbital. We complete this analysis by looking at the Se_2 atoms and splitting the contribution of the atomic states (see Figure S24). We can see from there that the p_z orbital dominates the valence band maximum around the Γ point, while the p_y contributes the most to the second to last valence state. This observation points to the direction of a certain insensitiveness of the valence band maximum to strain applied to both a and b direction (parallel to x and y in our case) as pointed out by our band structure calculations.

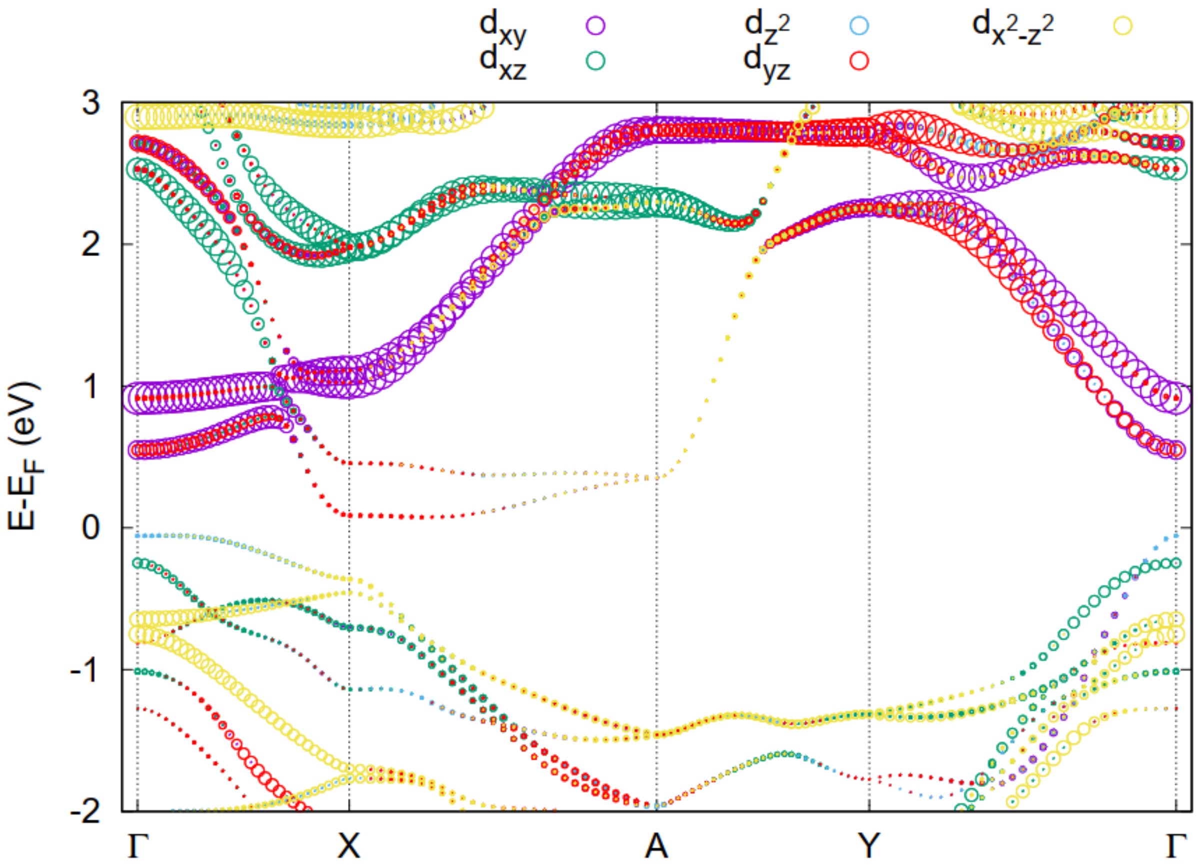

Figure S23. We thus project the bands into the contribution of the d orbitals of the Zr atoms. The s and p orbital do not contribute to this energy range.

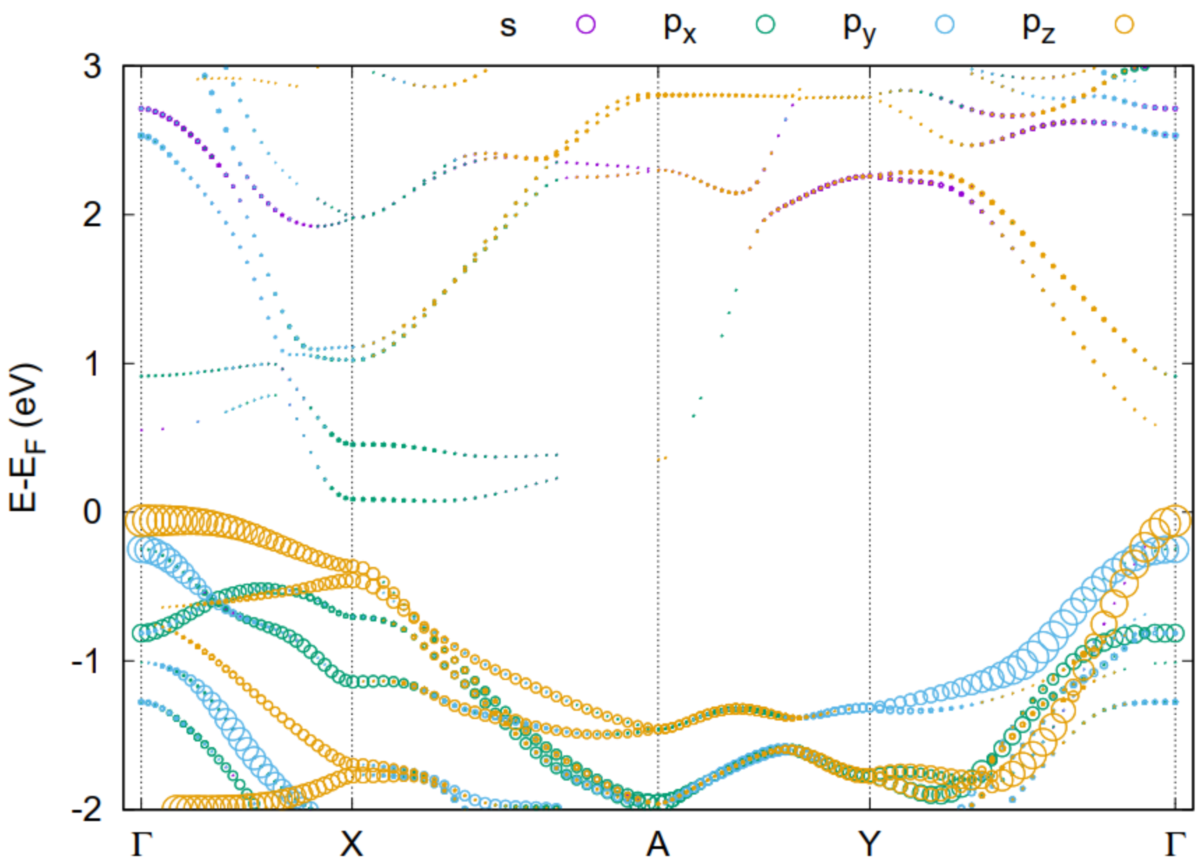

Figure S24. Band structure of $ZrSe_3$ projected along the s and p orbital of the Se_2 atoms.

Finally, in Figure S25, we plot the electronic density with different point of view: a) along the a-axis, b) along the b-axis. We can notice the anisotropic structure of the material. Electron density accumulate more between the Zr and Se atoms along the b axis rather than along a. This explains the different response of the system to the two uniaxial strains.

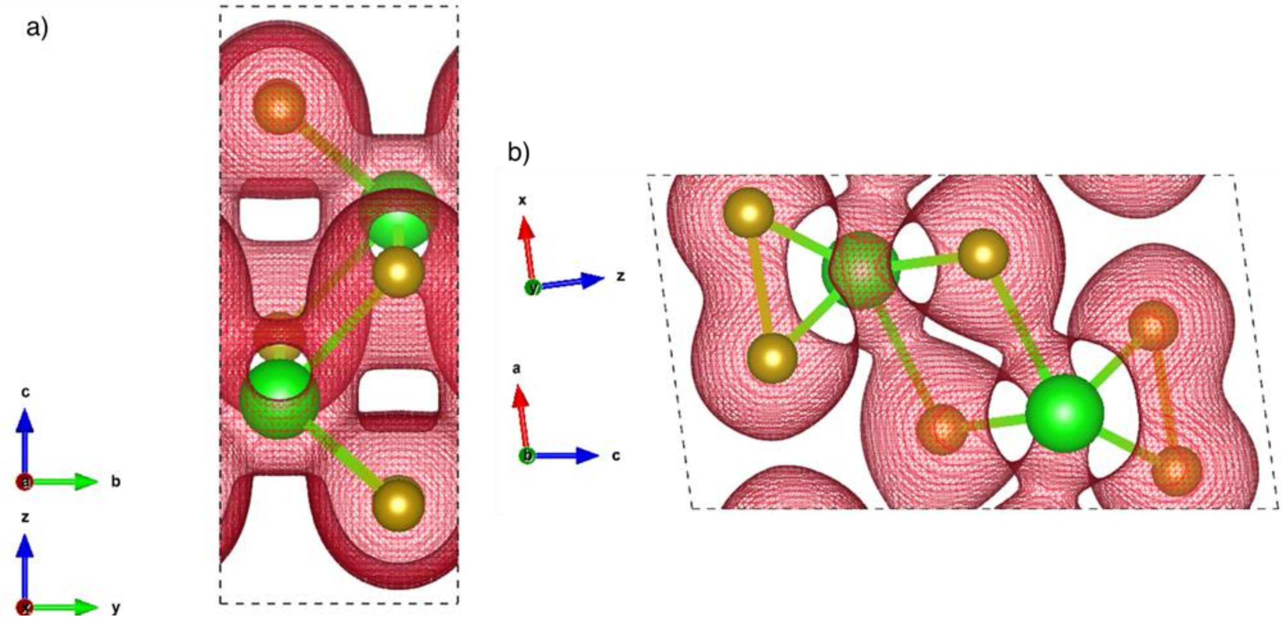

Figure S25. Electron density as projected along the a) a- and b) b-axis. The figure is obtained with VESTA [S3].

Supporting Information References

[S1] Costa, Pedro, Armando Ferreira, Vitor Sencadas, Julio C. Viana, and Senentxu Lanceros-Mendez. "Electro-mechanical properties of triblock copolymer styrene–butadiene–styrene/carbon nanotube composites for large deformation sensor applications." *Sensors and Actuators A: Physical* 201 (2013): 458-467.
[S2] Setyawan, W., & Curtarolo, S. (2010). High-throughput electronic band structure calculations: Challenges and tools. *Computational Materials Science*, *49*(2), 299–312.
[S3] K. Momma and F. Izumi, "VESTA 3 for three-dimensional visualization of crystal, volumetric and morphology data," *J. Appl. Crystallogr.*, 44, 1272-1276 (2011).
